# Functional acellular matrix for tissue repair

**DOI:** 10.1016/j.mtbio.2022.100530

**Published:** 2022-12-28

**Authors:** Bin Wang, Tang Qinglai, Qian Yang, Mengmeng Li, Shiying Zeng, Xinming Yang, Zian Xiao, Xinying Tong, Lanjie Lei, Shisheng Li

**Affiliations:** a Department of Otorhinolaryngology Head and Neck Surgery, The Second Xiangya Hospital, Central South University, Changsha 410011, China; b Department of Hemodialysis, The Second Xiangya Hospital, Central South University, Changsha 410011, Hunan, China; c State Key Laboratory of Bioelectronics, School of Biological Science and Medical Engineering, Southeast University, Nanjing 210096, China

**Keywords:** Decellularized extracellular matrix, Stem cell differentiation, Decellularized scaffold, Regenerative medicine, Tissue engineering

## Abstract

In view of their low immunogenicity, biomimetic internal environment, tissue- and organ-like physicochemical properties, and functionalization potential, decellularized extracellular matrix (dECM) materials attract considerable attention and are widely used in tissue engineering. This review describes the composition of extracellular matrices and their role in stem-cell differentiation, discusses the advantages and disadvantages of existing decellularization techniques, and presents methods for the functionalization and characterization of decellularized scaffolds. In addition, we discuss progress in the use of dECMs for cartilage, skin, nerve, and muscle repair and the transplantation or regeneration of different whole organs (e.g., kidneys, liver, uterus, lungs, and heart), summarize the shortcomings of using dECMs for tissue and organ repair after refunctionalization, and examine the corresponding future prospects. Thus, the present review helps to further systematize the application of functionalized dECMs in tissue/organ transplantation and keep researchers up to date on recent progress in dECM usage.

## Highlights

• The process of tissue repair using decellularization techniques is described in detail.
• Research advances in different areas of extracellular matrix decellularization are summarized.
• The achievements of extracellular matrix decellularization in tissue repair in recent years are summarized.

## Graphical abstract

**Figure:**
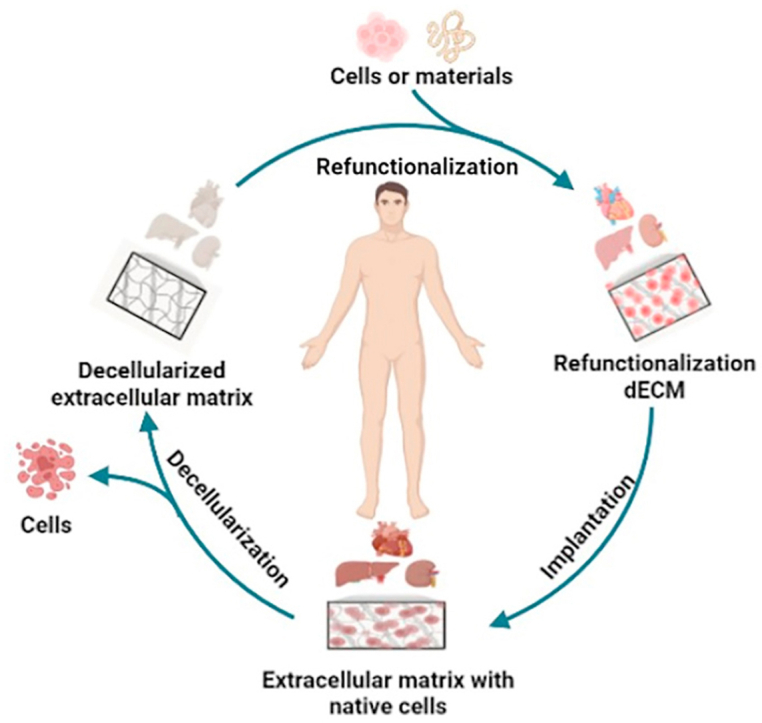

## 1 Introduction

Despite the great social and economic progress, our society still suffers from diseases causing serious tissue and organ damage, which highlights the importance of developing tissue repair technologies. A traditional method of tissue repair is autologous or allogeneic tissue transplantation [1]. However, autologous transplantation causes additional pain and injury to patients and suffers from the limited availability of donor sources [2], while the application of allografts (used to treat patients with donor tissue or organs) is restricted by the associated risks of immune rejection and disease transmission [3]. In view of the above, regenerative medicine, especially tissue engineering, has gained much attention, as it is considered to be the most promising strategy for tissue and organ repair [4].

When employed as matrix scaffolds for regenerative therapy, naturally derived biomaterials are superior to artificial polymers and have been used to construct complex matrices ranging from microtissues consisting of combinations of individual proteins to organ scaffolds generated through the decellularization of entire tissues. Organ/tissue-derived decellularized extracellular matrices (dECMs) feature the properties of a perfect tissue scaffold, namely a unique tissue-specific structure, complex vascular network, and composition, and are therefore well suited for both *in vitro* and *in vivo* regenerative medicine [5]. dECMs have been intensively studied since Poel completed the first exploration of their role in 1948 [6]. In the work of Badylak et al. (1995), small tears in the Achilles tendon of dogs were repaired using decellularized submucosa from the porcine small intestine [7], while Ott et al. (2008) first reported the decellularization and recellularization of an entire heart [8]. Macchiarini et al. (2008) extracted and decellularized the trachea of a cadaver and used chondrocytes derived from its epithelial and mesenchymal stem cells to repair the trachea of a patient with airway stenosis [9], while Basonbul and Cohen (2017) realized the endoscopic repair of the tympanic membrane in children using decellularized porcine small intestine submucosa [10]. ECM signaling influences organizational cell adhesion, migration, recruitment, differentiation, and proliferation based on the mechanism of (biological) tissue functioning [11]. These guiding elements allow one to retain at least partial functionality even in the absence of living cell components [12]. For this reason, dECM-based regenerative medicine methods for repairing damaged tissues or organs are being increasingly studied and applied in tissue engineering [13].

**Fig. 1:**
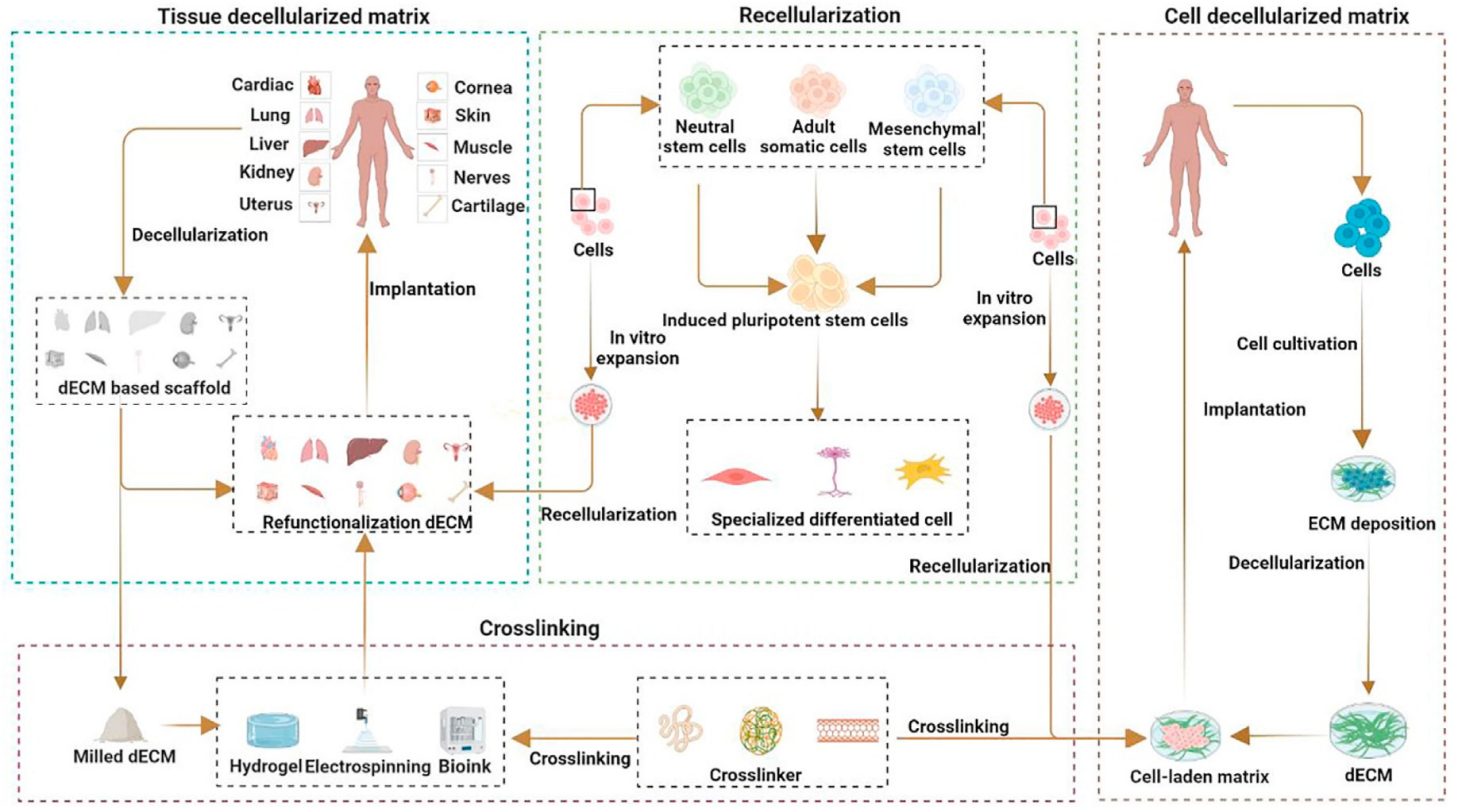
Schematic diagram of a functional decellularized extracellular matrix for individual tissue repair. Adapted reprinted with permission from Ref. [16], based on CC BY-NC-ND License.

The recent years have witnessed a series of studies in all directions of dECM usage, as exemplified by tissue source expansion, de- and recellularization protocol optimization, the search for more suitable crosslinking materials, and the use of functionalized dECMs for tissue and organ repair. However, progress in the field of functionalized dECM research in different human tissues and organs is inconsistent because of their complexity. For example, several dECM products (e.g., Allderm® and OASIS®) have been successfully translated into clinical applications [14], whereas functionalized renal dECM scaffolds are still unable to perform basic kidney functions *in vivo* [15]. In this review, we make a review of all the topics concerning ECM, from its composition and characteristics, through protocols for obtaining dECM, as well as its characterization, and finally ending with an extensive description of application examples. This paper can be easily read by researchers, many of whom are newcomers to the subject, and can help them quickly understand the field of dECM. We also present the main differences between cell- and tissue-derived decellular matrices and discuss their functions in stem cell and tissue repair, respectively. Meanwhile, this paper updates some emerging decellularization methods and summarizes their benefits and drawbacks, as well as some progress in the application of dECM in tissue repair, allowing experts in this field of dECM to have quick access to the most recent news. We also outline in the review some of the dECM products that have been commercialized, which are rarely mentioned in other similar articles. Finally, we also summarize some of the issues that still need to be addressed in this area of dECM and deal with future dECM applications in tissue engineering. The application of the functional decellularization of the extracellular matrix to tissue repair is schematically illustrated in Fig. 1.

## 2 ECM and dECM overview

### 2.1 ECM composition and function

The ECM is a distinct tissue-specific three-dimensional (3D) environment made up of structural factors and substances released by residing cells [17]. The cells interact with the ECM, with cellular products such as proteases, growth factors, and cytokines acting as functional cues to control cellular metabolic and secretory activities [13]. ECM proteins, which are broadly classified into fibrin and glycoproteins [18], regulate protein complexes, transmit cellular signals, bind growth factors, aid cell adhesion, and may also have other specific functions depending on the structure [19,20].

**Fig. 2:**
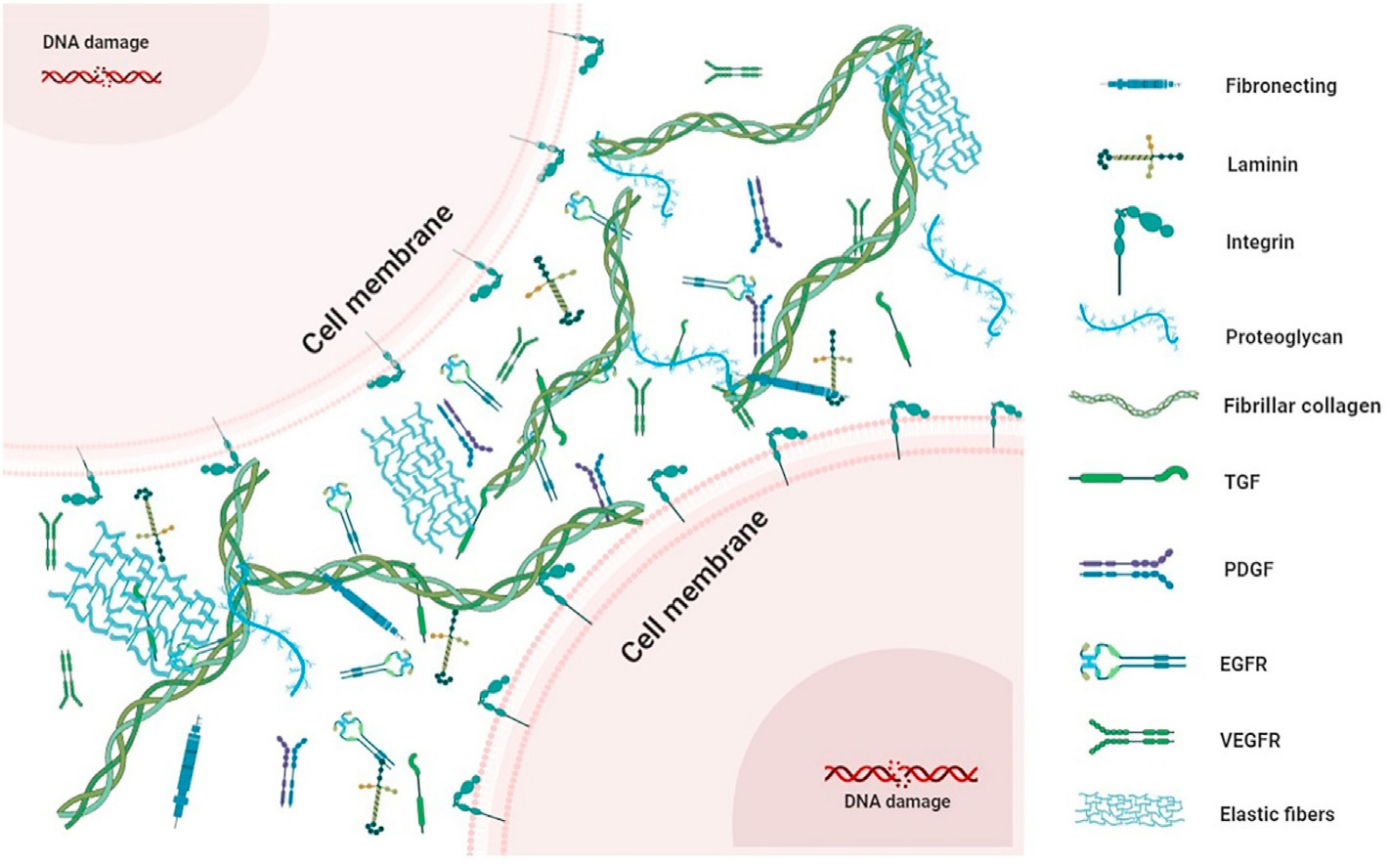
The extracellular matrix contains a variety of proteins and growth factors. Proteins are broadly classified into two major groups: fibrin (such as collagen, fibronectin, laminin, fibrillar, and laminin); and glycoproteins (such as proteoglycan). Growth factors, such as transforming growth factor (TGF), platelet-derived growth factor (PDGF), epidermal growth factor receptor (EGFR), Vascular Endothelial Growth Factor Receptor (VEGFR).

In addition to providing a structural foundation for tissue development, the ECM plays other important roles [21], e.g., is involved in tissue differentiation and organ isolation, establishment, and maintenance by regulating the essential growth factor and receptor hydration levels and the pH of the surrounding microenvironment [20,22], thereby influencing local cell behavior during cell migration, adhesion, differentiation, proliferation, and apoptosis [21]. Moreover, the ECM is involved in mechanical force transmission, signaling, and growth factor release [23]. Thus, the ECM is a highly complex tissue-specific structure and a repository of functional proteins capable of remodeling intrinsic cells and directing cell phenotype, survival, and behavior [21]. The composition of the ECM is illustrated in Fig. 2.

### 2.2 Association between ECM and stem-cell differentiation

Nearly all tissues, including blood and bone marrow, contain stem cells [5]. *In vivo* physical factors associated with stem-cell direction [24] include cell shape, external mechanical forces, and the ECM. The ECM is known to mechanically and chemically signal stem cells, which, in turn, influence the ECM by releasing growth factors and proteases into the same [25,26]. As a result, stem cells and the ECM are causally related in a reciprocal manner [27,28]. For instance, the dECM derived from adipocytes and bone marrow cells has a unique microenvironment with variable biomolecular structures and mechanical characteristics [29]. More significantly, this tissue-specific milieu affects stem-cell behavior in a variety of ways, e.g., by affecting stem-cell growth, morphology, and susceptibility to adipogenesis or osteogenesis induction [29]. In particular, osteogenesis is favored on harder or more viscous substrates, while adipogenesis is favored on softer substrates [[30], [31], [32]]. Therefore, the ECM can be both an inactive support and significantly impact stem-cell differentiation as a crucial component of stem cell ecology [33]. The relationship between the ECM and stem cell differentiation is shown in Table 1.

**Table 1:** Role of ECM in inducing stem-cell fate.

Role	Mechanism(s)	Function(s)	Ref
structural assistance	cell–matrix communication, mechanical properties, Porosity	Regulating cell proliferation, differentiation, and three-dimensional tissue architecture formation.	[22]
biochemical control	Integrins	Controlling cell ​homing, adhesion, ​migration, ​and differentiation.	[[34], [35], [36], [37]].
growth factor control	Sequestration, Gradients，activation, paracrine, reservoir, autocrine	Controlling the dynamic bioavailability of growth factors and preserving stem cell self-renewal, differentiation， and survival.	[[37], [38], [39]].
biological-mechanical control	Elasticity, stiffness, and microstructure ​of the ECM	modifying cell shape, tissue elongation, interactions between cells and the ECM, and controlling stem-cell destiny.	[37,[40], [41], [42], [43], [44], [45]].

### 2.3 dECM description and classification

dECM scaffolds are biomaterials formed from human or animal organs/tissues by removing immunogenic cellular components using decellularization techniques [47] and have several advantages over conventional stent materials [11,48,49]. For example, the 3D ECM structure remains intact after the removal of cellular and nuclear materials and preserves the natural ECM components for cell adhesion, proliferation, and differentiation. In addition, dECM stent materials contain cytokines and some signaling molecules in the natural matrix, e.g., Vascular Endothelial Growth Factor, basic fibroblast growth factor, and transforming growth factor-β, which can enhance cell function and promote vascularization. Furthermore, decellularization reduces ECM immunogenicity, allowing the use of tissues and organs of allogeneic origin and expanding the applicability of donated material for transplants. Finally, the dECM is biodegradable and does not induce inflammatory reactions. Similar to the ECM, the dECM mainly contains fibrin and glycosaminoglycan (GAG) [50]. Given that the physicochemical signals and biological properties of the ECM are retained after decellularization, the dECM can be used as a mechanical substrate and 3D biological carrier for subsequent cell inoculation [51,52]. The functionality of the dECM obtained from different tissue types is determined by the various growth factors, adhesion polypeptides, GAG, and collagen contained therein [53,54].

dECM scaffolds are classified as tissue-specific ECM (TS-ECM) and cell-derived ECM (CECM) [46]. TS-ECM is derived from decellularized tissues or organs from homologous or heterologous sources, such as cartilage, nerve, muscle, or heart tissue. CECM is obtained by culturing autologous cells or stem cells under aseptic conditions *in vitro*. CECM is obtained in a shorter and gentler manner than TS-ESM [55,56]. In addition, CECM scaffolds are more easily fabricated by using different types of cells, whereas with TS-ECM scaffolds, patient-specific cells such as mesenchymal stem cells, osteoblasts, chondrocytes, fibroblasts, and other cell types are required. The CECM scaffold can be prepared using cell-derived ECM particles, substrates, and *in vitro* scaffold-free live cell sheet culture systems. It can be used as a scaffold to maintain the desired geometry, bioelasticity, porosity, and biomechanical properties to enhance seed cell adhesion, differentiation, and proliferation, as well as to accelerate repair of damaged tissue [[57], [58], [59], [60]]. TS-ECM also has similar biocompatibility. It has been reported that cartilage-derived TS-ECM is more likely to promote chondrocyte differentiation, whereas C-ECM supports chondrocyte/stem cell proliferation and promotes chondrogenic potential, and that these C-ECM, alone or in combination with other factors, have varying abilities to promote chondrogenesis *in vitro* and *in vivo* [61]. In experimental animal studies and clinical trials, TS-ECM has been found to have some potential for pathogen transfer, inflammatory or anti-host immune responses, uncontrollable degradation, and other problems [[62], [63], [64]]. In contrast, the CECM stent can largely avoid these disadvantages. It can eliminate pathogen transfer to maintain a sterile matrix while providing the desired geometry and porosity and avoiding the limitations caused by poor cell permeability. In terms of applications, the CECM is often, but not always, used for the coating of biological materials, as exemplified by its application as a two-dimensional (2D) structure to promote wound healing or regeneration in bioengineered tissues [65]. In contrast to the TS-ECM, which is the perfect scaffolding substance for tissue engineering, the CECM is usually a place where primary culture cells or mesenchymal stem cells (MSCs) can be regenerated while maintaining their differentiation and potential for proliferation [61,66,67]. The CECM can regenerate bone marrow-derived MSCs [68,69], neural precursor cells [70], and periodontal ligament stem cells [71] and can enhance the differentiation and proliferation potential of primary cells such as chondrocytes [72,73], myeloid cells [74,75], and hepatocytes [76]. This ability is mainly due to anti-inflammatory and antioxidant effects [73,77], although the underlying mechanisms require further investigation. The TS-ECM exhibits the properties of a perfect tissue scaffold, namely a unique tissue-specific structure and complex vascular network and composition, and has two applications. First, the TS-ECM can be directly transplanted into the recipient, depending on his/her self-repair ability. Second, certain initiating materials can be added upon the implantation of a dECM scaffold to enhance its repair ability [78]. The dECM formed using initiating materials is also denoted as refunctionalized dECM. Currently, several functionalized dECMs such as porcine heart valves [79] and porcine small intestine submucosa have been approved for clinical use by the Food and Drug Administration of the United States [[80], [81], [82], [83]]. Thus, the functionalized dECM is a very promising material for tissue and organ repair.

## 3 dECM fabrication, characterization, and functionalization

**Fig. 3:**
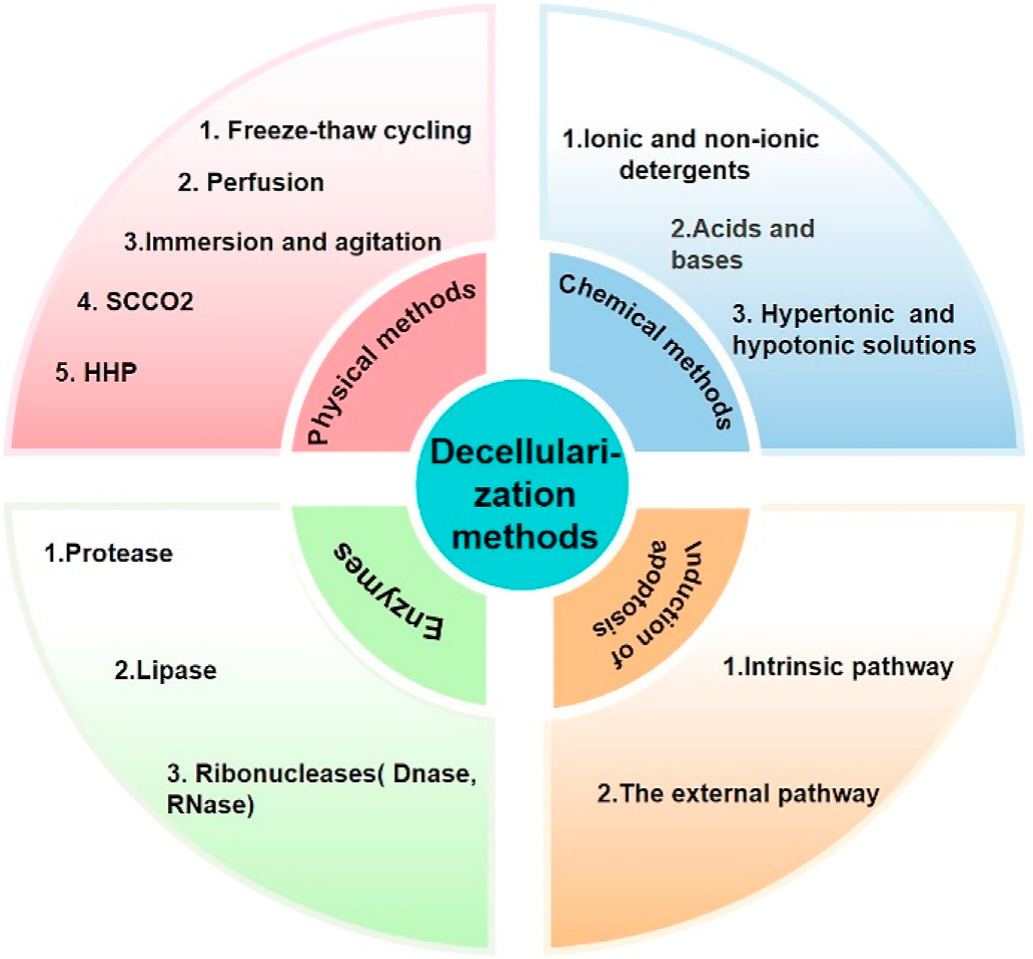
Methods of decellularization: chemical methods, physical methods, enzymes, and apoptosis.

In the past decades of decellularization research, various methods have been developed to obtain the dECM while preserving its biochemical composition, mechanical integrity, 3D structure, and biological activity and minimizing the possibility of immunological resistance [21]. However, there is no perfect decellularization solution, and the employed method heavily depends on the properties of the primary tissue of origin, e.g., age, site, and size [84,85]. Numerous decellularization protocols are available, including traditional strategies of disrupting the outer cell membrane by physical, chemical, and enzymatic methods, all of which remove cellular components but inflict certain structural and compositional damage [86]. Therefore, the combined use of several methods can maximize the emptying of cellular contents while minimizing the adverse effects on the ECM [[87], [88], [89]]. Although new decellularization protocols such as apoptosis [90,91] and vacuum-assisted decellularization [[92], [93], [94]] have been proposed, they are currently not widely used because of their complex mechanisms [86]. A sketch of standard and newly emerged decellularization methods is shown in Fig. 3.

### 3.1 dECM fabrication

#### 3.1.1 Physical methods

Physical decellularization methods rely on the disruption of the cell membrane under the action of force, pressure, or temperature [46], with common physical methods as well as their advantages and disadvantages discussed below.

Effective decellularization can be achieved using freeze–thaw cycling between −80 ​°C and 37 ​°C [95]. This method is commonly used for the decellularization of ligaments, tendon tissues, and nerve tissues [96] and has been applied to both the TS-ECM and CECM to improve decellularization efficiency, preserve tissue structure, biomechanical properties, and biochemical composition, and reduce cytotoxicity and chemical residues [97,98]. However, as the resulting membrane and intracellular components are still present after freeze–thaw cycling, subsequent processing is required to eliminate cellular residues [11,96]. Decellularization efficiency usually depends on variables such as cellularity, ECM density, and tissue thickness [21] as well as on the freezing/thawing rate, setting temperature, processing time, and number of cycles [99].

Perfusion is a typical physical decellularization method that involves the cannulation of an organ/tissue followed by the formation of a channel for the flow of a circulating agent through the intrinsic vascular system [100]. For large thick tissues and whole organs, this method minimizes ECM damage inflicted by excessive forces while maximizing decellularization deep inside the organ [5]. The decellularization efficiency of perfusion depends on organ type, perfusion route (arterial or intravenous), mode, and parameters (flow rate, pressure, and temperature), and perfusate composition and kind [87,[101], [102], [103], [104]]. Perfusion-induced decellularization can be highly efficient [[105], [106], [107]] while preserving the rich and complex vascular network [[108], [109], [110]] and has been applied to complex organs such as lungs, liver, kidneys, and heart [111]. However, perfusion is a very complex process requiring additional hardware and sophisticated flow control equipment such as pressure receptors and infusion pumps [[112], [113], [114]].

In another physical method, the tissue is immersed into a decellularization solution under continuous mechanical agitation. The effectiveness of this method depends on parameters such as agitation intensity, decellularizing agent, and tissue size [115,116]. Immersion under agitation is considered the best physical decellularization method [86] because of its high efficiency and short exposure time [113,117] as well as simplicity and ability to preserve the ECM surface structure, collagen structure, mechanical strength, and GAG content [107,[117], [118], [119], [120]].

Newer methods include nonthermal irreversible electroporation, which uses electrical pulses as an alternative to cell lysis and can disrupt cell membranes but is only applicable to small tissues [46]. In addition, supercritical CO_2_ has been used for bone decellularization [95]. These methods can significantly reduce decellularization time and improve disinfection compared to traditional methods [48]. Effective decellularization requires rapid decompression, and the high hydrostatic pressure used to disrupt cell membranes during decellularization can also kill viruses and thus eliminate the need for further sterilization [95]. Nevertheless, physical methods alone cannot achieve effective decellularization and may require subsequent treatment to eliminate membranous and intracellular residues generated in the tissue [21].

#### 3.1.2 Chemical methods

Chemical agents effectively remove cellular contents by promoting the hydrolytic degradation of biomolecules, disrupting cell membranes, and dissolving the bonds of inter- and extracellular junctions [11,96]. The main chemical decellularization methods use ionic or nonionic decontaminants, acids or bases, and hypertonic or hypotonic solutions [21].

Ionic decontaminants are synthetic organic compounds used primarily to remove genetic material and break down cell nuclei in various tissues and organs (e.g., bone) [95]. Sodium dodecyl sulfate (SDS) has found numerous applications as an ionic decontaminant [121] but may reduce the content of GAG and growth factors in the ECM [95] and is somewhat toxic [122]. Nonionic decontaminants (e.g., Triton X-100) are biodegradable emulsifiers that are used to solubilize proteins and degrade cell membranes [123], cleaving protein–DNA and lipid–protein bonds [124] while maintaining the integrity of protein–protein bonds [46]. The acids commonly used in decellularization include peroxyacetic, hydrochloric, and acetic acids [125], while the corresponding bases include sodium hydroxide, sodium sulfide, and calcium hydroxide [126]. Acidic compounds promote hydrolysis by forming covalent bonds or providing H^+^ ions [125], while bases function by inducing cellular cleavage and denaturing genetic material [86]. Hyper-/hypotonic solutions are those with solute concentrations higher or lower than intracellular concentrations, respectively, and are used to remove cellular components at the extracellular level. Hypertonic solutions can remove proteins, whereas hypotonic solutions can remove nuclei and DNA [127]. However, neither of these solutions can completely eliminate cellular residues [128].

Even though chemical agents can effectively achieve decellularization, they damage ECM composition and structure. Therefore, chemical decellularization protocols should be optimized for different tissues in combination with other decellularization protocols to minimize ECM damage.

#### 3.1.3 Enzymes

Enzymatic decellularization is highly specific and removes cellular components and unwanted ECM components by disrupting cell–matrix bonds or specific bonds in cells [86], with typical enzymes corresponding to trypsin, collagenase, lipase, dispase, thermophilic proteases, and nucleases [11]. When used in the first step of decellularization, trypsin can completely eliminate nuclei while preserving GAG content [129,130], which is essential for maintaining the biomechanical structure of the ECM [95]. However, studies on the effects of trypsin on collagen and elastin are limited [21]. Nucleases, including ribonuclease (RNase) and deoxyribonuclease (DNase), break down nucleotide sequences during cell lysis [131,132] and are therefore commonly used to remove nucleic acids after physical pressure and chemical decontamination agent–induced cell lysis [133]. However, prolonged nuclease action can lead to the loss of ECM components [118,134,135]. Dispersase can prevent cell aggregation [136]. Lipase is also used in decellularization, as it catalyzes the hydrolysis of lipid macromolecules [11,96]. The effective and relatively rapid removal of lipids from tissues can be achieved using treatment with alcohols such as ethanol and propanol [11,96], which, however, may lead to tissue clouding [11].

Although enzymatic decellularization protocols are highly specific, they may affect the structure and function of the native ECM and are not applicable to thicker tissues.

#### 3.1.4 Apoptosis

Apoptosis, i.e., genetically programmed cell death [137,138], proceeds via extrinsic and intrinsic pathways [12], both of which are driven by extremely complex and incompletely understood molecular processes. During apoptosis, the cell membrane undergoes structural changes resulting in the loss of contact between the cell and the ECM [139]. In addition, as the cell contents are strictly maintained within apoptotic vesicles and the cell membrane [140], their immunogenic material does not leak into the surrounding ECM [[141], [142], [143]]. Based on this, a novel decellularization scheme was developed for the controlled activation of apoptotic pathways via the delivery of appropriate signals [12]. For example, the extrinsic pathway can be activated by the specific ligands of the tumor necrosis factor superfamily death receptor, which alter the environment the cells are exposed to (e.g., temperature, pH, and CO_2_/O_2_, NO, and H_2_O_2_ levels). The intrinsic pathway can be activated by altering environmental stress or gene editing to make cells naturally undergo apoptosis.

Both approaches induce natural apoptosis by regulating the expression levels or implementing the toxic transgenes of key involved genes. The apoptosis method achieves decellularization with little change in matrix function or structure. The advantages and disadvantages of decellularization methods are summarized in Table 2.

**Table 2:** Advantages and disadvantages of decellularization methods.

Methods		Advantages	Disadvantages	Ref
Physical methods	Freeze-thaw cycling	Simple operation low demand for equipment. Cryoprotectant usage can minimize ECM disruption.	Require more treatments to get rid of the contents of the cells. Ice crystals' disruption of the ECM microstructure.	[99,[144], [145], [146]]
	Perfusion	Maximizing the delivery of decellularization deep inside the organ improves decellularization efficiency.	Stringent tissue requirements need for blood vessels. Perfusion is more complex and requires additional hardware and sophisticated flow control equipment.	[[117], [118], [119],[123], [124], [125], [126]]
	Immersion and agitation	The process is simple. Proteins of ECM can be well preserved.	Need the right mixing strength and time-consuming long.	[119,[129], [130], [131],^147^]
	SCCO2	Significantly reduce the decellularization time and improve disinfection compared to other methods.	Rapid decompression is necessary. Not widely used.	[71]
	HHP	Eliminating the need for further sterilization. Processes	Make it challenging for solutions to enter the ECM because of the constant high pressure.	[105,[148], [149], [150], [151], [152]]
Chemical methods	Ionic and non-ionic detergents	Versatile and suitable for a wide range of tissues.	Reduction of active ingredients in ECM Some toxicity	[105,134]
	Acids and bases	Cheap and less time-consuming	Damage to the ECM architecture, affecting the contents of the ECM.	[[153], [154], [155]]
				
	Hypertonic and hypotonic solutions	Hypertonic solutions can remove proteins, whereas hypotonic solutions can remove nuclei and DNA.	Unable to completely eliminate cellular residues.	[98,140]
Enzymes	Protease	Less time-consuming	Difficult to intern adequate decellularization. Affecting the contents of the ECM. Not suitable for sensitive tissues	[141,156]
	Lipase	Help the process of decellularization by first eliminating the epithelium and endothelium.	Hard to remove all lipids	[[156], [157], [158]]
	Ribonucleases (Dnase, RNase)	Effective removal of DNA levels from dECM	Easily residual, difficult to completely eliminate reagents Influencing the structure of ECM	[127,159,160]
Induction of apoptosis	Intrinsic pathway	The structure and function of dECM remains almost unchanged.	Apoptotic pathways are very complex and not yet fully understood. Not widely used.	[12]
	The external pathway			

Abbreviations: ECM: mExtracellular Matrix. dECM: decellularized extracellular matrix. SCCO2:supercritical CO2.

### 3.2 Characterization of dECM

Any decellularization step can change ECM composition, structural properties, and integrity, thus affecting the biological and mechanical properties of the resulting dECM [21]. In addition, undesirable products may be released during decellularization [161], and residual cellular components may lead to cytocompatibility problems and cause adverse reactions in the host [22]. Therefore, the careful characterization of dECM properties is a task of high importance. The following minimum criteria for competent decellularization quantification were proposed [11]: (1) <50 ​ng of dsDNA per mg of ECM dry weight; (2) DNA fragment length <200 bp; and (3) lack of visible nucleated material in 4′,6-diamidino-2-phenylindole (DAPI) or hematoxylin and eosin (H&E) -stained tissue sections. These criteria primarily focus on the characterization of DNA removal, as residual DNA is responsible for most adverse host reactions [159,162]. Common characterization techniques include protein composition assays of the dECM, residual assays of cells, observation of general macroscopic structures such as the vascular system and pore size [8,163,164], and biomechanical and structural analyses.

#### 3.2.1 Microscopic techniques

Microstructure assessment methods include those relying on light microscopy, which has been employed for preliminary qualitative research on tissue (e.g., cornea) structure [157]. Phase-contrast microscopy allows the optical path to be easily modified to increase image resolution and contrast [157]. Collagen tissue may be measured quantitatively, and polarized light microscopy can spot any structural alterations in collagen that might take place during decellularization [157]. Detailed *in vivo* observation is made possible by confocal microscopy [157]. Electron microscopy is mainly used to provide local information on tissue structures [21]. Transmission electron microscopy (TEM) has greater resolving power than light microscopy for ultrastructural feature analysis and is a core technique for gaining insights into natural tissue structures, including the order, diameter, and spacing of collagen fibers [157]. Scanning electron microscopy (SEM) provides lower resolution than TEM and is mainly used to characterize the sample surface and morphology and thus determine the cellular morphology of tissues [157]. Atomic force microscopy (AFM), which uses a piezoelectrically controlled fine tip to scan the tissue surface [157], can measure the interstitial areas between collagenous protofibrils (unlike SEM and TEM) and is used for the nondestructive screening of decellularized tissues and TEM data validation [157]. The second-harmonic imaging of the cornea was reported to provide high spatial resolution and contrast comparable to those achieved by light and electron microscopy imaging [165]. High-frequency ultrasound (50 ​MHz) was reported to provide higher (up to 30-μm) resolution than traditional ultrasonography methods [166].

#### 3.2.2 Proteomic analysis

The in-depth analysis of dECM proteins is a task of high importance, as they determine the bionomic properties of the dECM [21]. Gel electrophoresis separates proteins according to their molecular weight, thus effectively solving the problem of mixing various protein components in the dECM [167]. The combination of mass spectrometry (MS) and 2D gel electrophoresis is effective for screening proteins and peptides in tissues or organs [21]. MS methods commonly applied to decellularized materials include liquid chromatography tandem mass spectrometry [167], time-of-flight secondary-ion mass spectrometry [168], and matrix-assisted laser desorption/ionization mass spectrometry [169]. Messenger RNA microarrays allow the examination of proteins adsorbed on the dECM surface [170]. However, the available evidence suggests that tissue proteomic analysis based on protein extraction may induce the disruption and dissociation of ECM proteins to varying degrees depending on tissue type and donor age; therefore, advanced strategies are needed to confirm the tissue specificity of the prepared dECM [21].

#### 3.2.3 Cell residue determination

The most common methods used to assess cellular material removal and residuals are histology, DNA staining, and imaging [157]. Routine histological staining and immunofluorescence are used for qualitative verification to prove the efficient removal of nuclear contents and the cytoplasm [21]. The initial step of evaluation often corresponds to H&E staining, in which case hematoxylin is most frequently used to determine the degree of decellularization, while eosin is typically used to evaluate the composition of the nonnuclear ECM [157]. Saffron, Movat's pentachrome, and Masson's trichrome are additional histological stains enabling the qualitative detection of various extracellular and cytoplasmic components [96]. DNA staining for detecting cellular and nuclear components as well as fluorescent DAPI, Hoechst, and PI staining can be used to detect residual DNA [21]. In addition, the terminal Deoxynucleotidyl Transferase mediated dUTP Nick-End Labeling (TUNEL) method is used to assess possible apoptosis during decellularization by detecting the number of DNA fragments based on the fluorescent labeling of nucleic acid ends [157]. DNA staining and imaging are common ways to assess decellularization results and are often used as first-line studies. However, these methods are usually not very accurate and do not give quantitative data. Immunochemical techniques such as toluidine blue, Alcian blue, and Verhoeff-van Gieson staining are frequently employed to identify dECM components [157,171].

#### 3.2.4 Quantification of residual chemicals

Unfavorable residues in decellularized cells can cause severe immunological reactions and hinder recellularization [172]. Therefore, the quantification of residual chemicals after dECM preparation is an essential task. For example, methylene blue binding assays have been used, along with the quantification of residual SDS in decellularized cruciate ligaments by the collagenase-induced digestion of supernatants prepared from scaffolds [173]. Visible-light spectroscopy is another simple method of quantifying SDS in decellularized cells [170]. Residual SDS has also been quantified by gas chromatography, and 6 ​h was suggested to be the optimal wash time for significantly reducing the content of residual SDS in decellularized liver scaffolds [174]. The presence of residual decellularization chemicals is a major problem, as they can be cytotoxic even at low concentrations [21]. Therefore, it is important to develop suitable decellularization protocols and methods for quantifying residual chemicals to achieve a trade-off between retaining sufficient ultrastructural features and effective proteins of the ECM and simultaneously removing as much cell content as possible and minimizing the amount of residual chemicals.

#### 3.2.5 Biomechanical and structural analyses

The balance between effective decellularization and the sufficient preservation of the ECM structure is a major challenge of organ/tissue decellularization. By correlating the results of mechanical analysis after decellularization with the retention degree of natural ECM structures, one may understand the role of proteins in determining ECM biomechanics [96,175]. Therefore, tissue tensile strength and elasticity can be used to evaluate ECM preservation. Specific tests are used for applications in tissue engineering, as exemplified by (i) swelling tests, in which the entire tissue or film is inflated through a window in the substrate, and its displacement is measured and correlated with mechanical strength [176,177]; (ii) pressure tests, in which the material is compressively deformed between two plates under a known load to determine its resilience during fragmentation [178,179]; and (iii) uniaxial tensile tests used to measure the elastic modulus [171]. Despite the availability of various methods, the mechanical characteristics of biological structures remain challenging to measure, especially under sterile conditions [157]. The test results are difficult to compare because of the wide variation in the technical setup, and the interpretation of the results can lead to discrepancies in data-derived mechanical properties [180].

#### 3.2.6 Disinfection

Disinfection is required to lower the possibility of negative immunological reactions [21]. Standard clinical sterilization methods, including the application of pressurized steam, dry heat, or chemicals, inevitably result in protein denaturation [181]. Other sterilization agents such as electron beam irradiation, gamma irradiation, and ethylene oxide can change the mechanical and ultrastructural characteristics of the dECM [182,183] and impact the functionality of dECM-containing clinical products [184,185]. Disinfection with antibiotics significantly inhibits bacterial growth by disrupting bacterial cell walls and preventing the production of DNA and proteins while having an insignificant impact on decellularized scaffold structures [86]. However, each antibiotic has a limited antibacterial spectrum [186,187]. Compared to other disinfection techniques, treatment with supercritical CO_2_ can be used as a substitute for sterilization and induces less variation in the mechanical properties of the ECM [188]. Nevertheless, further research is required to confirm whether sterilization can be achieved without destroying the dECM.

### 3.3 dECM refunctionalization

#### 3.3.1 Crosslinking

Crosslinking, which can be performed physically or chemically, is often used to preserve the 3D structure of the dECM, improve scaffold characteristics [189], and reduce inflammatory potential. Given that the decellularization process usually adversely affects ECM characteristics, the dECM is usually strengthened by crosslinking to obtain modified dECM. For example, tissue decellularization using 1-ethyl-3-(3-dimethylaminopropyl)carbodiimide (EDC) crosslinking combined with chemical extraction can promote the adhesion and differentiation of MSCs [190]. In a recent report, acrylate groups were transferred to a decellularized platform by photocrosslinking to enhance the mechanical properties of the dECM and form hydrogels with high shape stability [191]. In addition, the methylene blue–mediated photo-oxidative crosslinking of a tumor dECM greatly increased the stiffness of the scaffold but hardly changed the amide III band of the peptide and protein secondary structures [192]. The chemical modification of gelatin to afford gelatin methacrylate (GelMA) improved the adherence and homing ability of bone marrow MSCs in the ECM [193]. A GelMA hydrogel with functionalized ECM was used to repair irregular cartilage defects. In addition, dECM crosslinked with hyaluronic acid maintained the natural collagen secondary structure and the microporous structure of the porcine decellularized dermal matrix, exhibited enhanced degradation resistance and moisturizing ability, and promoted wound healing [194]. Methacrylic acid, hyaluronic acid, and gelatin were used to successfully regenerate mature cartilage *in vitro* and in an autologous goat model, affording a characteristic trap structure and cartilage-specific ECM [195]. The crosslinking effect of glutaraldehyde, oxidized chitosan oligosaccharide, and carbodiimide on the dECM of bass significantly improved the mechanical properties and degradation resistance of the acellular dermal matrix, which indicated that carbodiimide can improve matrix properties and has potential applications in biomaterial engineering [196]. In another study, rat decellularized lung was bend into tannic acid crosslinked tissue (TA-CLT) or EDC/*N*-hydroxysuccinimide (NHS)-crosslinked tissue (EDC/NHS-CLT) [197]. TA-CLT strongly induced T-cell proliferation and attenuated macrophage proliferation, while EDC/NHS crosslinking provided physical attributes similar to those of natural lung tissue. These studies indicate that the original biological properties of the dECM can be recovered (or even improved) by crosslinking with various reagents, which plays an important role in the reduction of the corresponding inflammatory potential. However, the possible toxic effects of crosslinking agents should not be ignored.

#### 3.3.2 Recellularization

Recellularized scaffolds are those that have had various cell types (e.g., stem cells, chondrocytes, and epithelial cells) implanted on them to impart form and function [198]. For example, tracheal cartilage was successfully regenerated and repaired using photocrosslinked hydrogels combined with chondrocytes [191]. Stem cells are usually classified into pluri- and multipotent ones, among which the former are more capable of differentiation and include induced pluripotent stem cells and embryonic stem cells [86]. MSCs can differentiate into various cell types and show remarkable performance in the field of tissue engineering, as exemplified by tendon repair, bone regeneration, cardiomyogenesis, and skin wound healing [[199], [200], [201], [202], [203], [204], [205], [206], [207]]. In view of its biological and physical characteristics, the dECM is considered a suitable biological scaffold for the application of MSCs in tissue repair, making MSCs differentiate at specific sites and working in concert with cells to heal tissues [207]. In the case of decellularized corneas, recellularization strategies are classified into (1) the *ex vivo* inoculation of constructs for the downstream transplantation of cellularized grafts and (2) *in vivo* implantation, which enables the graft to be repopulated with host cells following surgery [157]. Recent studies have dealt with the repair of damaged tissues/organs using cell-free strategies, that is, the activation of cells and their accumulation at damage sites induced by appropriate stimulation and recruitment factors, the recruitment of endogenous stem cells to the damaged sites, and tissue repair [208]. However, these factors require further investigation. Furthermore, the recellularization of decellularized substrates is affected by many factors such as the diversity of cell types, recellularization method, cell density, and culturing conditions [86].

## 4 Tissue repair using functional dECMs

### 4.1 Cartilage and bone regeneration

Currently, Bone defects caused by trauma, tumors, or osteoarthritis remain challenging [209,210], two biomaterial types are primary used in cartilage tissue engineering, namely (1) synthetic biomaterials such as polylactic acid [211] and polycaprolactone [212] and (2) natural biomaterials such as fibrin [213], gelatin [214], and collagen [215]. Owing to the complexity of cartilage ECM, natural scaffold or biomaterial-derived ECM holds great promise for cartilage repair [13]. Both macro- and microstructural characteristics are preserved in dECM scaffolds, which greatly improves osteoconductivity [[216], [217], [218]]. Various dECM scaffolds have been used in bone and cartilage repair, including injectable hydrogels and electrospun and 3D printed scaffolds.

**Fig. 4:**
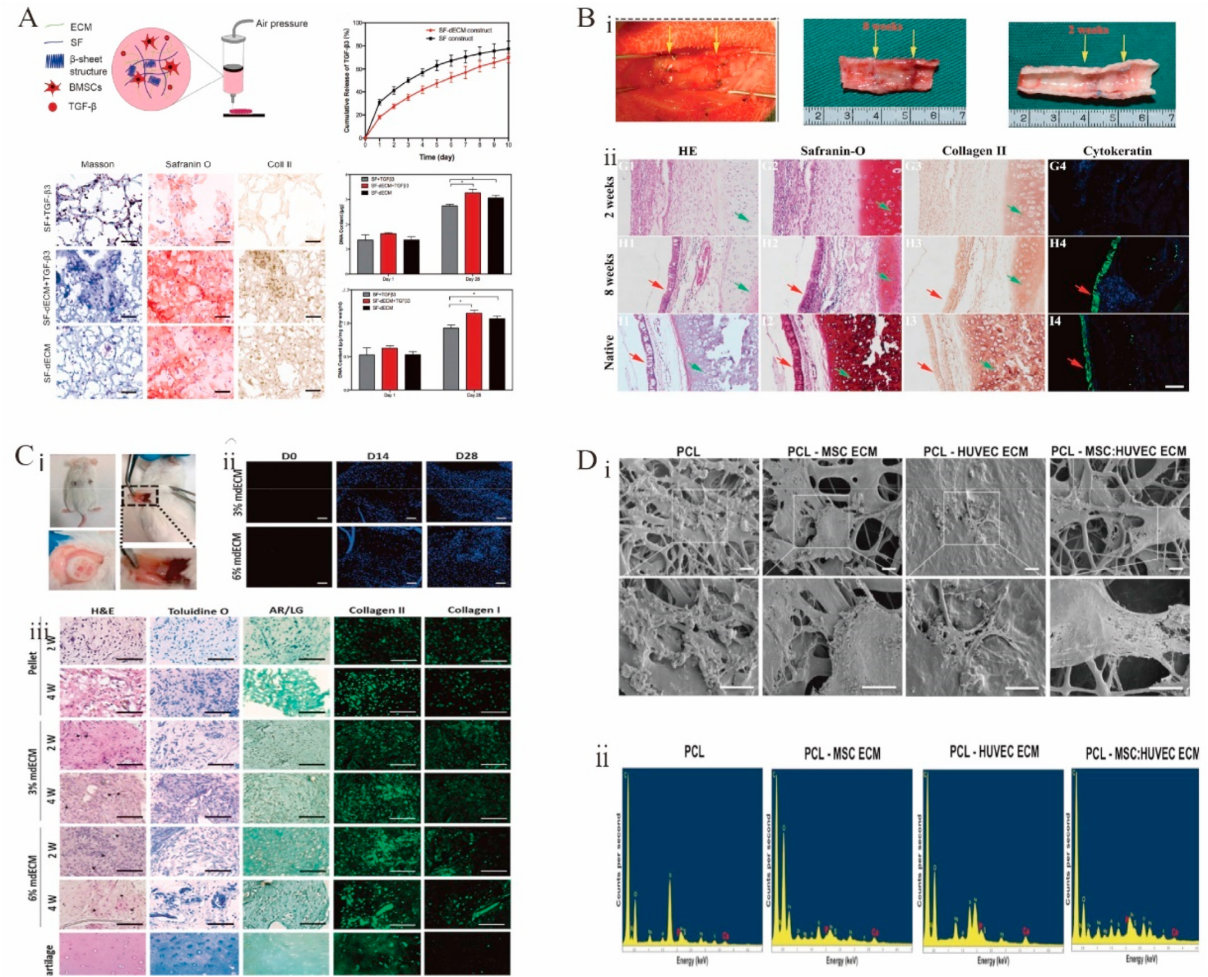
Functional decellularized extracellular matrix in Cartilage and bone repair. (A) These findings demonstrated the ability of the SF-dECM 3BDP scaffolds to encourage chondrogenesis and cartilage regeneration *in vivo*. Adapted reprinted with permission from Ref. [237](License number:5,442,250,848,007). (B) Application of engineered tracheal cartilage for tracheal reconstruction. (i) Engineered tracheal images at the time of surgery, 2 weeks postoperatively, and 8 weeks postoperatively. (ii) Epithelialization was not evident at postoperative week 2, but by week 8, the epithelial layer was visible and not significantly different compared to that of the natural gas tube. Adapted reprinted with permission from Ref. [191], (License number:5,442,390,855,450). (C) Biological evaluations of mdECM hydrogel *in vivo*. (i) Pictures taken four weeks after implantation in CD1 mice. (ii) With time, a significant increase in the number of nuclei was seen. (iii) mdECM hydrogel acquired the typical shape of chondrocytes embedded in lacunae after 2 weeks *in vivo*, which was more evident in the highest concentration. Using toluidine O staining, it was evidenced that the deposition of GAGs had increased. Adapted reprinted with permission from Ref. [221] (License number:5,442,400,012,079). (D) MSCs have differentiated into osteoblasts. (i) After 21 days of osteogenic development, SEM scans showed calcified nodules. (ii) After 21 days in culture, electro spun scaffolds contain calcium and phosphorus. Adapted reprinted with permission from Ref. [229] (License number:5,442,400,497,756). Abbreviation：SF-dECM: silk fibroin and decellularized extracellular matrix, mdECM: from mesenchymal stem cells.

Hydrogels based on dECM have been extensively investigated for cartilage and bone regeneration. Hydrogel has good absorption, satisfactory biocompatibility, and high safety [219]. Bioadhesive hydrogels show great potential for bone regeneration [220]. For example, MSCs were used to prepare well-biocompatible bionic hydrogels inducing chondrogenesis and further hyaline cartilage formation without the addition of induction agents (Fig. 4C) [221]. However, the storage modulus of hydrogels made of bone dECM (∼150 ​Pa at 6 ​mg/mL) was lower than that of bone (8–11 ​GPa) [222], which affected hydrogel mechanical properties. The binding of cartilage ECM particles modified with the affinity peptide sequence PFSSTKT to GelMA hydrogels enhanced the mechanical properties of hydrogels and allowed them to provide a good 3D supported microenvironment [193]. An acrylic anhydride–crosslinked dECM hydrogel facilitated the formation of homogeneous cartilage and the repair of cricoid tracheal injury in a rabbit model (Fig. 4B) [191]. The crosslinking of carbodiimide with GAG in the absence of pepsin improved the mechanical properties and increased the osteogenic capacity of dECM [223,224], which suggests that the use of an enzymatic treatment protocol during matrix decellularization may affect the biomechanics of the subsequent dECM scaffold and the retention of certain matrix factors. Different tissue origins may also be one of the factors influencing the induction of osteogenesis in dECM scaffolds. Some studies showed that dECM scaffolds derived from cartilage or adipose tissue are more osteogenic than those derived from lung or spleen tissue [224].

Electrospinning is widely used in tissue engineering [[225], [226], [227], [228]], as it produces fibrous and porous scaffolds with good interconnectivity, high porosity, and elevated surface area. These properties facilitate proliferation, cell attachment, waste exchange, and nutrient uptake [225,227], allowing one to mimic the hierarchically ordered fibrous structure and architecture of the ECM [229]. However, electrospun scaffolds cannot achieve biological functions owing to the lack of cellular activity. In contrast, dECM has good bioactivity, although its mechanical quality is insufficient for regenerating and supporting bone or other hard tissues [230,231]. Thus, the CECM can be combined with electrospun scaffolds to enhance their mechanical quality. CECM-polycaprolactone scaffolds [232] and Ti implants [233] have been used for bone healing with excellent cell proliferation and osteogenic differentiation. Carvalho et al. developed the first ECM–polycaprolactone electrospun scaffold cocultured with the ECM from MSCs/stromal cells and the ECM from umbilical vein endothelial cells, showing that the resulting material had excellent osteogenic properties and a promising future in bone repair (Fig. 4D) [229].

3D bioprinting is an emerging technology in the rapidly advancing area of cartilage tissue engineering and is mainly used to print dECM scaffolds for later cell growth [234]. Recently, 3D printing has been used to create “living” tissue structures by depositing live cells with printable biomaterials (“bioinks”) [235,236]. A bioink prepared using silk fibroin and dECM was mixed with MSCs for the 3D bioprinting of porous structures with high mechanical strength, appropriate degradation rate, and precise shape capable of supporting BMSC proliferation and promoting cartilage differentiation (Fig. 4A) [237]. However, the difficulty of preparing suitable bioinks and the toxicity of most chemical crosslinking procedures used for bioink fabrication hinder the widespread adoption of this technology.

CECM is used as a cell culture matrix in bone tissue engineering and regenerative medicine because of its good biocompatibility and biodegradability. CECM is rich in collagen and proteoglycans, which facilitate the proliferation and osteogenic differentiation of MSCs and maintain their multidirectional differentiation potential while having lower reactive oxygen species levels and higher bone morphogenetic protein-2 (BMP-2) sensitivity [238]. In addition, *in vivo* transplantation assays have shown that MSCs still have the ability to form large amounts of bone tissue after the expansion of additional generations on an ECM-based culture platform [239,240]. Sun et al. reported that CECM is more biased to support chondrocyte/stem cell proliferation and promote chondrogenic potential than dECM of cartilage tissue origin due to a number of microstructural (mean pore size and fiber diameter), micromechanical, insoluble (e.g., collagen and GAG), and soluble factors [61]. Some researchers, too, have developed hybrid scaffolds consisting of CECM and various types of inorganic materials. For example, Antebi et al. combined stromal CECM into a collagen/hydroxyapatite (COL/HA) scaffold and found that MSCs cultured on the cECM-Col/HA scaffold proliferated significantly faster than those cultured on the Col/HA scaffold, and the expression levels of the osteogenic markers alkaline phosphatase (ALP), bone bridging protein, and Runx2 were also higher than those of cells cultured on the Col/HA scaffold alone [241]. The construction of a hybrid scaffold using human lung fibroblast-derived decellularized ECM as a carrier and inoculation with human placenta-derived MSCs was found to induce more new bone formation and a more complete repair of bone defects [242]. This shows that CECM tends to promote the proliferation and differentiation potential of MSCs, improving their quantity and quality.

### 4.2 Skin repair

The skin consists of three layers, namely a compound squamous epithelium mainly composed of keratinocytes, a subcutaneous tissue containing fat, and a dense dermis with fibroblasts rich in ECM and containing extensive blood vessels, hair follicles, and sweat glands [243]. The loss of skin integrity may lead to severe physiological imbalance and subsequent injury [244]. Although skin damage can heal spontaneously [245], this self-healing capacity may be exceeded in cases of major trauma such as large or severe burns and skin wounds caused by chronic diseases such as diabetes. Under these conditions, autografting remains the standard of care [246,247] but is not suitable for large burns because of the limited availability of skin grafts and is less effective for treating diabetic wounds [248]. Diabetic wounds are one of the most common diabetic complications, and they are chronic and difficult to heal [249]. Growth factors are essential for regulating the cellular response to the wound healing process [250,251]. A variety of biomaterials and bioactive compounds have now been shown to be effective in wound healing [252]. A novel angiogenic 3D bioprinted peptide patch to improve skin wound healing [253]. Luckily, dECM-based biomaterials can improve the healing of diabetic wounds and increase the survival of third-degree-burn patients [243,254].

**Fig. 5:**
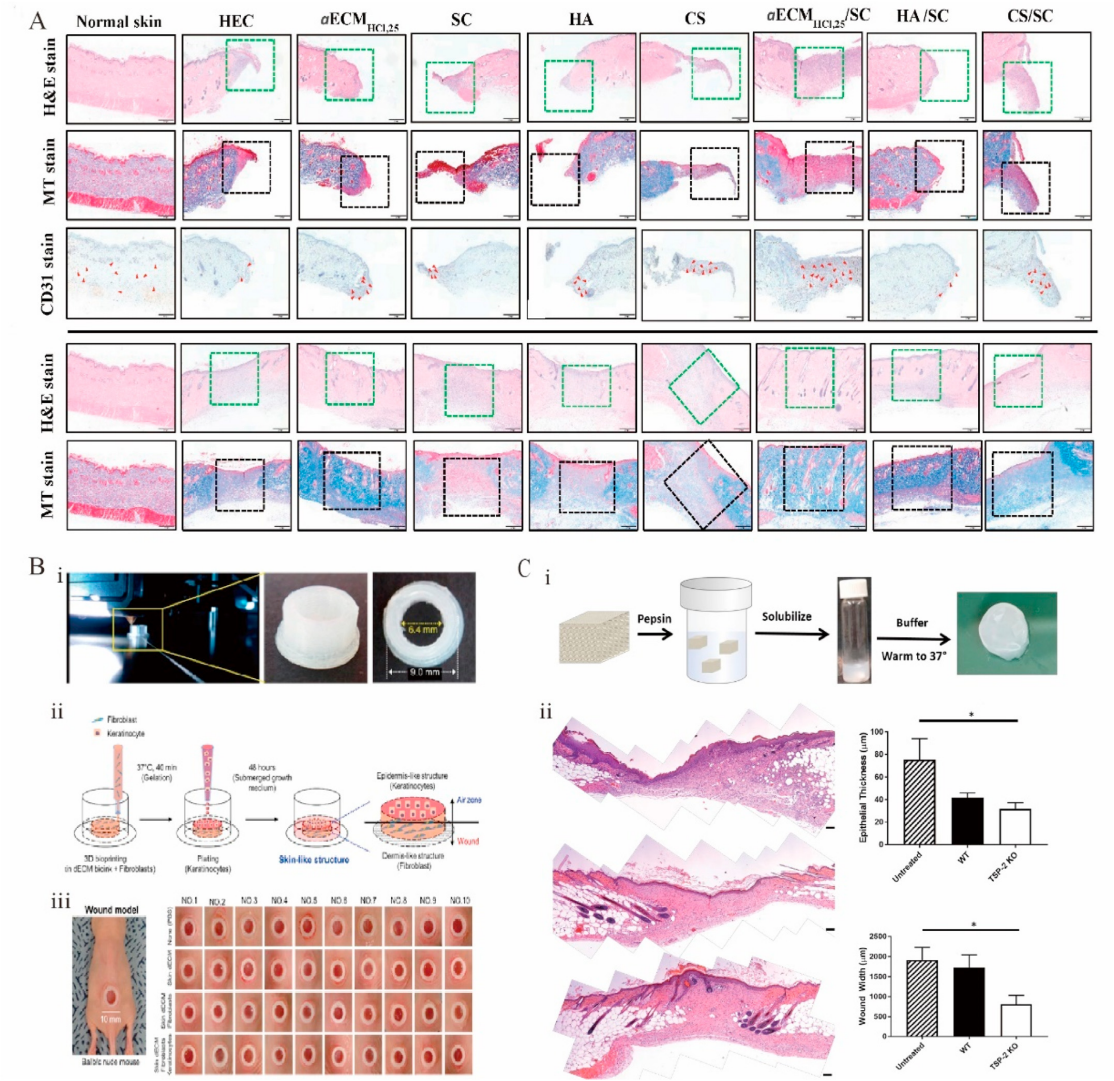
Functional decellularized extracellular matrix in the skin repair. (A) Histological analysis of normal skin and wounds treated with different dressings showed that the aECMHCl, 25/SC dressing promotes the healing of diabetic wounds. Adapted reprinted with permission from Ref. [255], based on CC BY License. (B) Skin substitutes and generation of chimney structures through 3D printing. (i, ii) Constructing chimney structures using a 3D printer and creating skin substitutes using 3D cell-printing technology. (iii) Validating uniform chimney model production in all experimental groups. Adapted reprinted with permission from Ref. [262], based on CC BY License. (C) Genetic manipulation allows for tissue-derived hydrogel repair of diabetic skin wounds. (i) A schematic diagram of the hydrogel preparation and example macroscopic images of the hydrogel (ii) Representative suture images of untreated, WT gel treated, or thrombospin-2 knockout gel-treated. Adapted reprinted with permission from Ref. [256]. Copyright © 2018 American Chemical Society.

In skin-repair applications, dECM-based hydrogels have been found to facilitate diabetic wound healing [248]. In one study, during the healing process, histological H&E staining (Fig. 5A) was performed on hydrogel dressings, and the results were compared with those obtained for normal skin on days 8 and 14. The wounds were closed on day 14 with no significant inflammation and a moderate number of fibroblasts occupying the dermis, which was considered skin wound healing [255]. A TSP-2 KO dECM hydrogel accelerated wound healing in diabetic mice by promoting wound angiogenesis and remodeling (Fig. 5C) [256].. Previous studies have mainly demonstrated the occurrence of revascularization and epithelialization during skin repair. A new composite hydrogel dressing containing a glycophorin and decellularized pepsin–formic acid–soluble ECM was shown to synergistically promote diabetic wound healing and help regenerate hair follicles and sweat glands [255]. Placenta-derived dECM hydrogels containing sulfated GAG were shown to effectively promote wound healing, which implies a combined biological function for ECMs containing sulfated GAG [257]. Several dECM products (e.g., Allderm® and OASIS®) have been successfully translated into clinical applications [14] but do not rebuild the hair follicle or sweat gland structure, which is the key factor for assessing skin repair. Some reports suggest that human placenta–derived dECM can regulate hair follicle formation [258,259].

CECM also offers great advantages in skin repair. An electrostatically spun fibrous membrane of l-propylene-co-caprolactone (PLCL) and human fibroblast-derived ECM (hFDM) was found to proliferate faster and exhibit a more elongated capillary-like morphology when human umbilical vein endothelial cells (HUVECs) were inoculated on hFDM-PLCL [260]. Tang et al. discovered that incorporating the extracellular matrix secreted by human adipose-derived stem cells into electrostatically spun poly nanofiber dressings improved wound healing in a surgically created total skin excision mouse model [261].

3D bioprinted dECMs have also been used for skin repair and regeneration. A skin-derived dECM bioink mimicking the microstructure and bioactivity of skin more closely than previously developed homogeneous bioinks was reported [243]. Porcine-derived dECM has been used as a bioink to produce artificial skin tissue by 3D printing. Synthetic skin was 3D printed using bioinks based on porcine skin, human dermal fibroblasts, and keratin-forming cells with dECM and evaluated for skin wound healing using a mouse wound model (Fig. 5B) [262]. The artificial skin was revealed to be a suitable skin substitute for the treatment of burns and autologous skin grafts. The application of dECM-based 3D bioprinting provides a new strategy for printing full skin layers, including structures (e.g., gland and hair follicles).

### 4.3 Cardiac repair

Heart disease can cause serious harm to patients [263]. dECM stents mimicking the natural heart environment hold great promise for cardiac therapy [264,265]. The dECM is used in various forms for cardiac therapy and is generally used in the form of a solid scaffold for natural vascular system structures or as a soluble material that can be formed into injectable hydrogels for tissue repair [5,266].

**Fig. 6:**
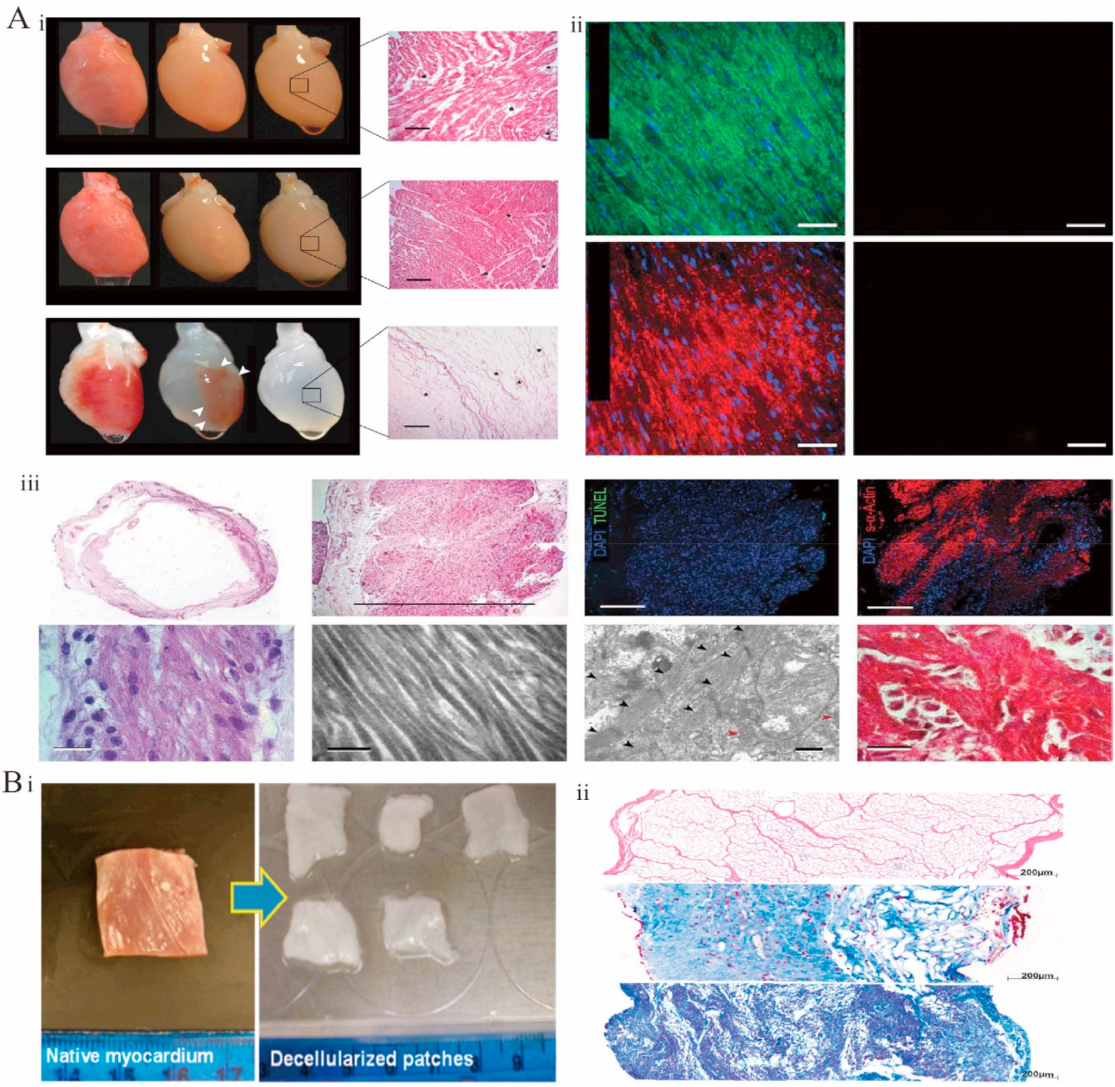
Functional decellularized extracellular matrix in the Cardiac repair. (A) Whole heart perfusion decellularization experiment in rats. (i) Gross view of whole heart decellularization in rats, H&E staining, and immunofluorescence images. (ii)Sections of decellularized rat hearts stained with immunofluorescence did not detect nuclei or contractile proteins. (iii) Analysis of the histology and electron micrographs of recellularized rat heart constructs. Adapted reprinted with permission from Ref. [8] (License number:5,442,460,220,568) (B) Natural myocardial and decellularized myocardial scaffolds. (i)Morphology of natural myocardium and decellularized myocardial scaffolds. (ii)H&E staining of the stent after decellularization and the H&E staining of the stent after two weeks and four weeks of recellularization. Adapted reprinted with permission from Ref. [278] (License number:5,442,470,287,789).

Solid dECM scaffolds are directly used after decellularization without any further microstructure disintegration. This approach preserves specific dECM components, the natural tissue structure, and the vascular system [267]. Solid stents are classified according to their application as tissue-engineered dECM patches or whole hearts [267]. Perfusion-decellularized whole-heart stents preserve the 3D structure of the original heart, including the vascular system [[268], [269], [270]], chamber geometry, and valve function, which is crucial for the subsequent synchronized heart beating [8,271,272]. In 2008, Ott et al. reported the first-time decellularization and recellularization of an entire heart [8]. Specifically, a whole rat heart was decellularized by perfusion through coronary arteries using 1% Triton X-100 and SDS (Fig. 6A–i). The heart preserved the complex ECM composition, chamber geometry, and vascular structure (Fig. 6A–ii). In the following years, the method was extended to larger cardiac organs from pigs and humans to enable the development of human-sized heart grafts [163,205,[273], [274], [275]]. For example, a porcine decellularized whole heart was obtained by retrograde coronary perfusion [276]. The first decellularized scaffold of a whole human heart was prepared using perfusion [163], which preserved the 3D structure, heart chamber geometry, vascular system, and mechanical properties. In addition, after recellularization, the myocardial genes of the heart expressed electrical binding properties. Cardiac patches were shown to be implantable [267]. In a rat model, the ventricular function was similar to baseline values after decellularization from porcine patch treatment [277]. Cardiac dECM patches were developed (Fig. 6B–i), and the physical characteristics of the heart tissue were recovered by reinoculating the cells, with the resulting tissue showing angiogenic potential and cardiac regeneration functions (Fig. 6B–ii) [278].

Soluble dECM has a broad scope of application in cardiac repair, as it is suitable for preparing 3D and 2D hydrogels *in vivo* or *in vitro* and can be directly applied to the myocardium or added to other materials or cells to make cell-containing biologically active injectable gels or cardiac patches [279]. Some limitations of soluble heart dECM include the poor retention of heart structure and mechanical properties. Many research groups have tried to solve these problems and tune dECM properties by crosslinking or adding different materials. The results indicate that the mechanical properties, gelation, and degradation of the soluble dECM in the heart can be regulated using crosslinking agents, polymers, and matrix metalloproteinase–inhibiting drugs [[280], [281], [282], [283]]. In addition, 2D coating or hydrogel models are excellent platforms for assessing the cellular response to different dECM [267]. Soluble dECM niches have been used to induce cell–matrix binding, improve infarct environments, model bioecological niches and matrix rigidity, and evaluate the way dECM regulates stem-cell phenotype and matrix binding [267]. Soluble dECM is a flexible system for the development of composite biomaterial scaffolds that can be loaded with stem cells. The development of multimaterial scaffolds, 3D printed structures, and electrospun materials may promote the further application of dECM in cardiac repair.

### 4.4 Nervous system

The nervous system includes the peripheral and central nervous systems, with the central nervous system composed of the spinal cord and the brain [284] working together to produce and transmit signals. Neurons themselves have relatively low regenerative capacity, especially in the central nervous system. Currently, spinal-cord injury or stroke damage cannot be repaired using surgical methods [248]. Neural dECM biomaterials have received considerable attention due to their ability to guide the growth of neurons and axons, stimulate myelin regeneration in Schwann cell axons, and promote the differentiation of stem cells into neurons [[285], [286], [287]]. dECM stents feature an intact native structure that can guide cell migration and direct axonal trajectories [288,289]. In addition, preserved ECM substances such as GAG regulate synapse formation and affect stem-cell proliferation [290,291].

**Fig. 7:**
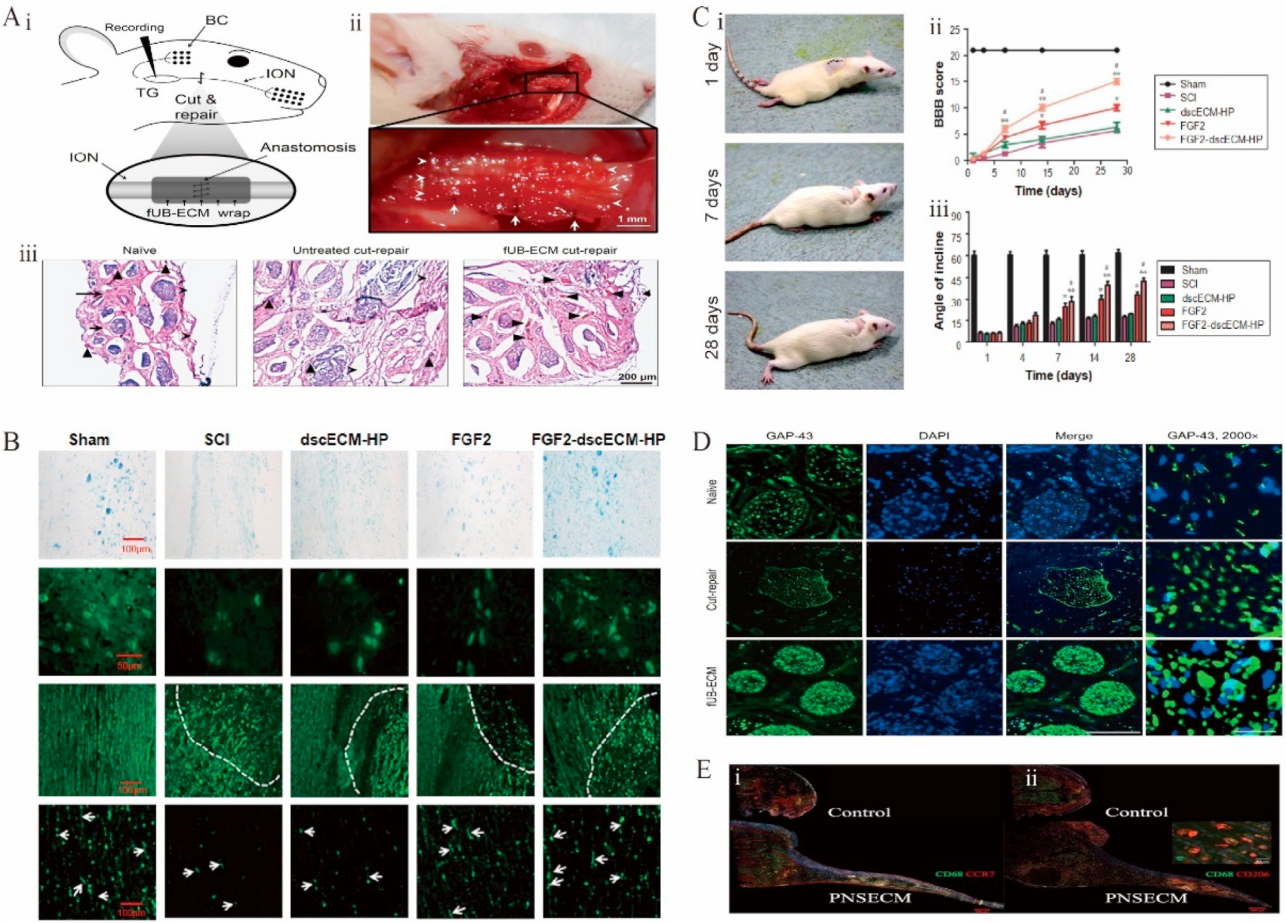
Functional decellularized extracellular matrix in nervous system repair. (A) Positive modulating effect of FUB-ECM on tissue remodeling after ION dissection repair. (i) Schematic of the ION cut and repair model showing the anastomosis and FUB-ECM nerve wrap. (ii) After 28 days, fUB-ECM nerve wraps remained sutured around the ION. (iii) H&E staining showed that in fUB-ECM cut-repair IONs, the organization resembled naïve IONs.Adapted reprinted with permission from Ref. [279], based on CC BY License. (B) ScI rat locomotor function assessments. (i) Movement of scI rats' hind limbs after FgF2-dscecM-hP hydrogel treatment, (ii) The Basso, Beattie, and Bresnahan scores, (iii) After various treatments, the scI rats' inclination angle [298]. (C) After 28 days of therapy, spinal cord-injured rats had nerve fiber healing and axonal regrowth. Adapted reprinted with permission from Ref. [298], based on CC BY License. (D) Growth-associated protein-43 expression is increased by FUB-ECM nerve wrapping during ION cut and repair. Adapted reprinted with permission from Ref. [279], based on CC BY License. (E) Insert shows surface CD206 labeling and intracellular CD68 labeling. In controlled portions, no further expansion of the proximal stump was seen. Adapted reprinted with permission from Ref. [293] (License number:5,442,480,428,926) Abbreviations:ION:infraorbital nerve. FUB-ECM: fetal porcine urinary bladder extracellular matrix. SCI: Spinal cord injury. FgF2:factor-2. DscecM: spinal cord extracellular matrix. hP：heparin-poloxamer.

Despite the certain regeneration ability of peripheral nerves, severe injuries usually require intervention [292]. In another study, fetal porcine-bladder ECM wraps were used to repair the infraorbital nerve transection of the trigeminal nerve in rats (Fig. 7A–i). This treatment significantly healed the outer and inner nerve tissue (Fig. 7A–ii) and increased the expression of growth-associated protein-43 and neovascularization (Fig. 7A–iii), as observed 28 days after surgery (Fig. 7D) [279]. However, whisker-evoked response properties and ionic axon remyelination were observed, and the number of neurofilament-positive axons remained unchanged, which suggested that improvements in tissue remodeling do not necessarily imply axonal regeneration or the recovery of mechanoreceptor cortical signals. Compared to controls, repair sites obtained using hydrogels with a peripheral nerve–specific ECM saw a tendency for macrophages to be distributed at the edges of regenerating bridges (Fig. 7E) [293]. CECM has also made progress as a cell culture substrate in neural tissue engineering. Gu et al. cultured dorsal root ganglion neurons from Sprague-Dawley rats at embryonic day 18 in Sherwan CECM and found that the axons of the neurons grew faster and were more divergent. In a follow-up study, they went on to combine Schwann CECM with chitosan catheters and filament fibers to form a hybrid scaffold that was implanted into the sciatic nerve interstitial site in rats, again showing enhanced regeneration of the sciatic nerve [294].

The ability of the central nervous system to self-heal after structural damage is not as well developed as that of the peripheral nervous system. Therefore, dECM stents for the central nervous system have received little attention [295,296]. Stents for the repair of spinal-cord injuries are currently derived from the spinal cord, sciatic nerve, paravertebral muscle, brain, optic nerve, and bladder matrices [284]. The MS analysis of dECMs derived from the sciatic nerve and spinal cord revealed that proteins associated with axonal growth and myelin regeneration, e.g., cohesin, are unique to the spinal cord, whereas some proteins such as Col IV α1 and α2 are unique to the sciatic nerve [297]. Sciatic dECM hydrogels were hypothesized to better promote axonal myelin regeneration in the spinal cord, whereas spinal dECM hydrogels were thought to better promote synapse formation. However, the rats did not show full recovery of hind paw mobility after the use of dECM hydrogels in a spinal-cord injury rat model. To investigate this issue, exogenous nerve regenerative growth factors (Fig. 7B), FGF-2, and a heparin poloxamer (Fig. 7C) were added to the spinal dECM hydrogel, and the rats regained movement to the extent of uninjured controls after treatment [298]. Nevertheless, effective methods for spinal-cord decellularization are not yet available, and existing ones rely on a combination of chemical, physical, enzymatic, and detergent techniques.

dECM-based therapies for brain injury have received little attention, as research has mainly focused on tissues from brain or urinary bladder matrices decellularized for use at injured brain sites [284]. A bioink based on a porcine brain dECM was used to develop patient-specific glioblastoma microarrays [299]. The brain dECM prevents neurological deficits caused by traumatic brain damage by reducing proinflammatory microglial cell responses, lowering glial scar formation, and improving the neurobehavioral function [300].

Although neuronal dECM biomaterials are partially successful in treating peripheral nerve injuries, no discernible effects have been observed for the treatment of central nervous system injuries [301]. Although nervous system repair has been extensively researched, there is still an urgent need to develop improved bionic scaffolds.

### 4.5 Respiratory organs

Although lung transplantation is currently the only treatment option for patients with advanced chronic obstructive pulmonary disease [248], its application is limited by a severe shortage of donor lungs [302]. Pulmonary tissue engineering focuses on two main methods, namely vehicle carriers for distal lung delivery and drug and whole-lung substitutes [303,304].

**Fig. 8:**
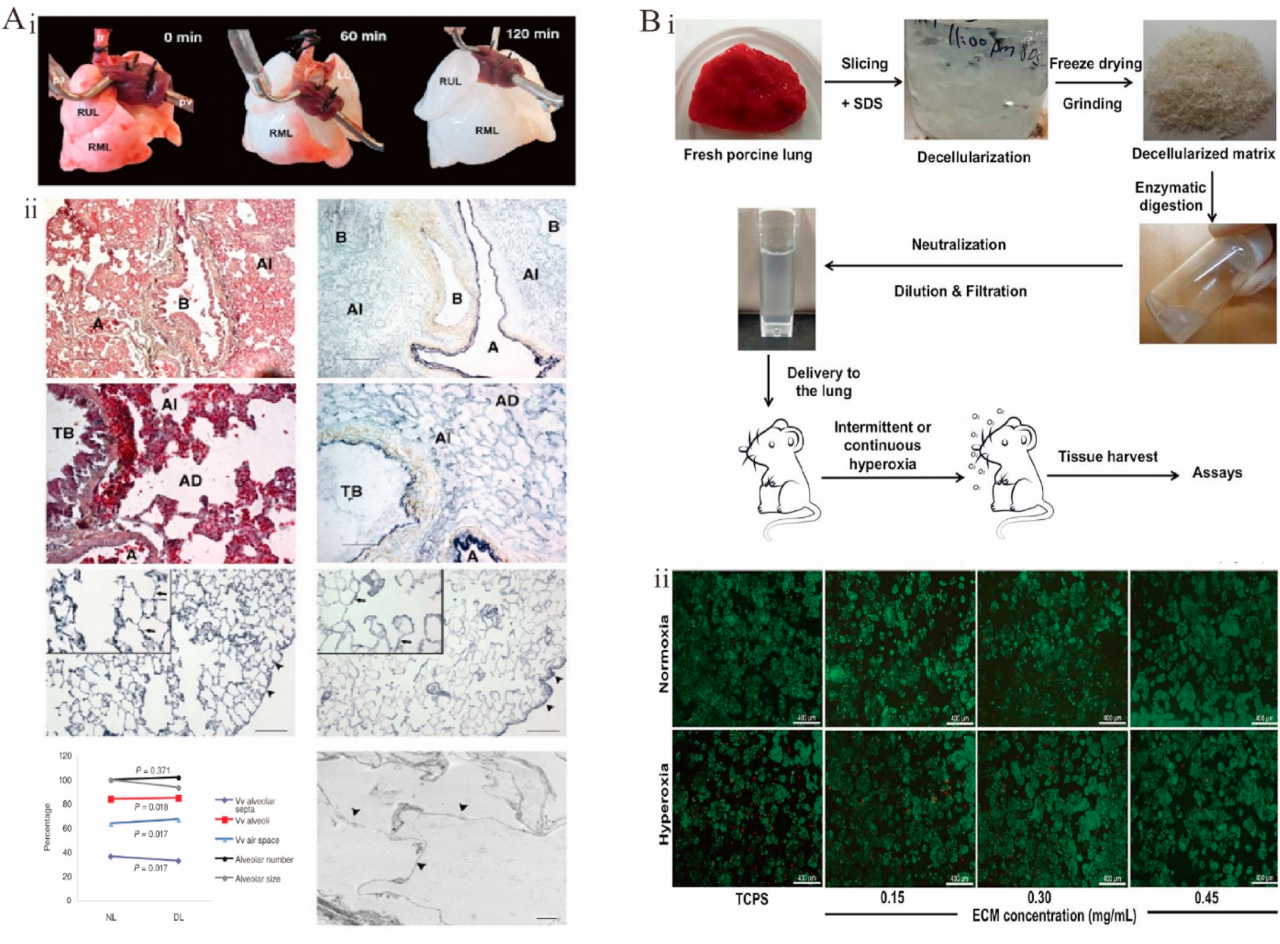
Functional decellularized extracellular matrix in respiratory organs repair. (A) Perfusion decellularization of whole rat lungs. (i) Photographs of a rat lung, mounted on a decellularization apparatus allowing antegrade pulmonary arterial perfusion. (ii) Corresponding Movat pentachrome and Verhoeff's elastic-tissue staining of thin sections from parenchyma of native (left) and decellularized (right) rat lung. Adapted reprinted with permission from Ref. [305] (License number:5,450,760,527,174). (B) Manufacture and evaluation of lung ECM solutions. (i) Lung ECM solution preparation and delivery. (ii) Live/dead staining images of Human lung epithelial cells on tissue culture polystyrene in mediums without lung ECM and with 0.15, 0.3and 0.4 ​mg/mL lung ECM under normoxia and hyperoxia. Adapted reprinted with permission from Ref. [316], based on CC BY License.

In 2010, Ott et al. used decellularization to produce a bioartificial lung (Fig. 8A–i), providing cell-free blood vessels, airways, and alveoli while preserving alveolar septa, alveolar surface area, and extracellular matrix proteins (Fig. 8A–ii) and enabling gas exchange both *in vivo* and *in vitro* [305]. In 2011, respiratory function was partially restored in rats with *in situ* transplanted recellularized lungs [306,307]. In 2017, transplanted artificial lungs were shown to facilitate gas exchange in a porcine model [308]. Recently, lung dECM–based 3D printing has been used to simulate lungs structure and function. For example, 3D printing was used to mimic the vascular and subsegmental bronchial structures of human lungs in an alginate-dECM lung composite [309]. The composite retained biological functions at multiple stages of tissue maturation, including the tissue-specific differentiation of primary human stem cells, regulation of *in vivo* immune responses, and vascularization after transplantation. The possibility of employing decellularized lungs as a natural 3D bioengineering matrix was established using a bioreactor system with a decellularized mouse lung matrix [310], and a model was provided for studying lung regrowth from stem cells and developing a rapid, controlled, and automated lung decellularization protocol [311]. Human CECM-active scaffolds with lung epithelial cells and primary fibroblasts from patients with non-chronic lung disease were used to create a unique cellular microenvironment for lung tissue engineering that demonstrated excellent bioactivity [312].

Another approach in lung tissue engineering is the use of drug carriers for distal lung drug delivery. Both dripping and nebulization are effective methods to deliver drugs to lungs [[313], [314], [315]]. Although coarse particles can be dripped through the tracheal tube, their distal lung distribution is inconsistent [314]. Nebulization encourages the distal lung ECM to be distributed uniformly, although the amount of ECM in the solution and the size of ECM microparticles are limited [316]. However, dripping and nebulization can reduce alveolar exudation and septal thickening. Decellularized ECM powder from bladder mucosa was demonstrated to alleviate bleomycin-induced pulmonary fibrosis when dripped into the lungs [317]. Lung-specific dECM microparticle suspensions/solutions for nebulization showed efficacy in minimizing lung oxidative injury. The nebulized lung dECM can act as both a mediator and a drug delivery vehicle of organ restoration. Ideally, the remodeled lung tissue should restore the mechanical properties of the lung (Fig. 8B) [316]. Despite significant advances in the fabrication of biomaterials for the lung dECM, this technology is still in its infancy. The functionalized lung dECM shows great promise for applications in the development of lung regrowth stimulation and lung disease models.

### 4.6 Digestive system

**Fig. 9:**
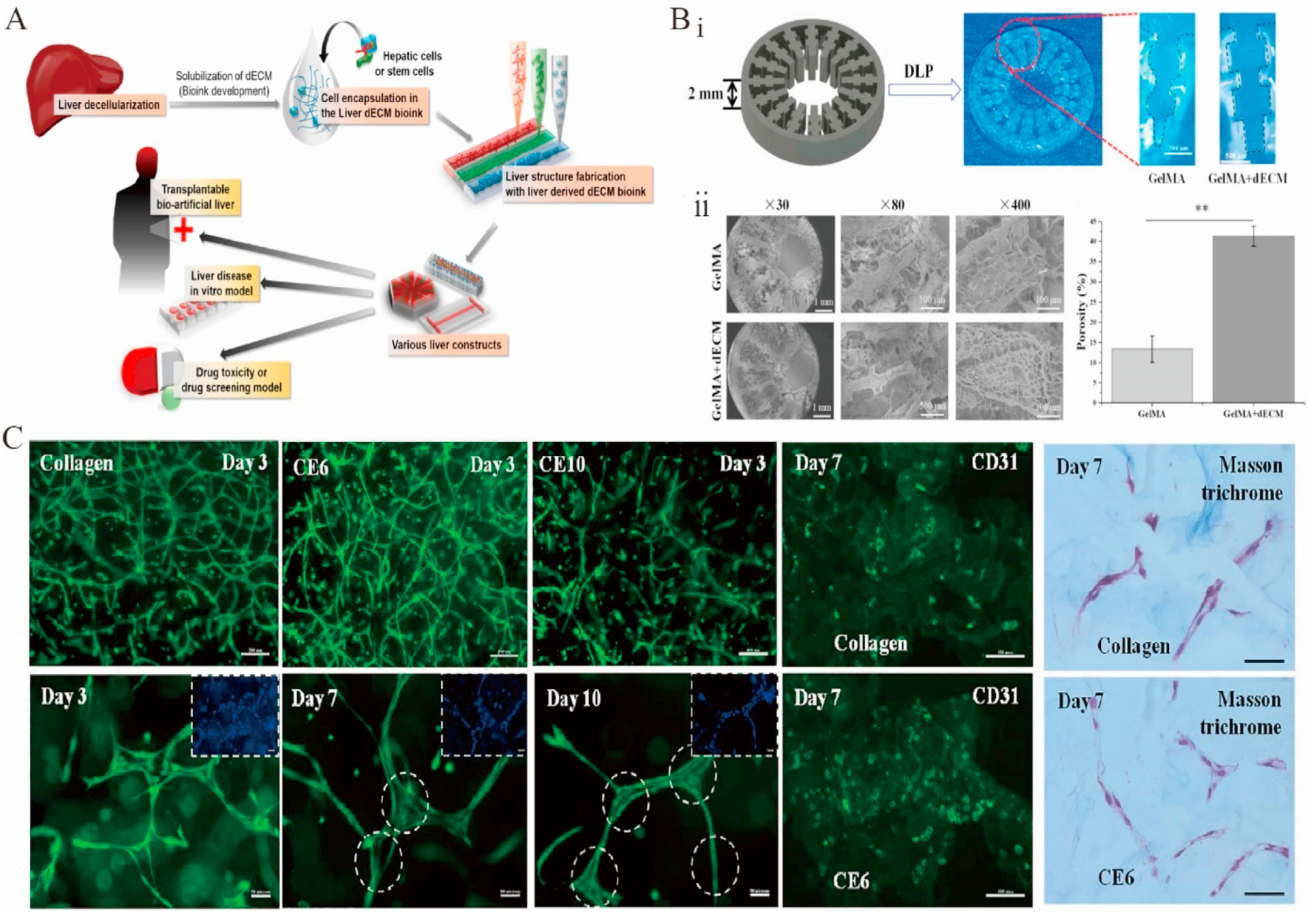
Functional decellularized extracellular matrix in digestive system repair. (A) Diagram of liver dECM bioink synthesis and use in liver tissue engineering via 3D cell printing. Adapted reprinted with permission from Ref. [321]. Copyright © 2017 American Chemical Society. (B) Macroscopic (i) and microscopic (ii) images of digital light processing printed (methacrylated gelatin) GelMA/dECM and GelMA scaffolds. Adapted reprinted with permission from Ref. [324](License number:5,450,770,224,374). (C) Decellularized Caprine liver ECM hydrogels for vasculogenesis assay. Adapted reprinted with permission from Ref. [322] (License number:5,450,771,384,453).

Liver diseases have attracted increasing attention. In 2010, Uygun et al. reported a new method of transplantable liver graft production using the effective recellularization of a decellularized liver matrix with human hepatocytes [318]. In 2011, a natural ECM scaffold for *in vitro* liver regeneration was developed by perfusing an entire liver with a natural liver vascular network detergent to delaminate cells [319]. In 2015, a human liver was decellularized and evaluated in terms of biocompatibility and quality [320]. In 2017, a liver dECM bioink inducing stem-cell differentiation and enhancing human hepatocellular carcinoma (HepG2) cell function was developed (Fig. 9A) [321]. In 2018, a hydrogel made of decellularized goat liver was shown to be effective in 2D/3D human hepatocyte and vascular endothelial cell culturing, featuring an increased potential to develop prevascularized liver structures for tissue-engineering applications (Fig. 9C) [322]. In another study on liver tissue engineering, extrusion printing was used to develop a thermally crosslinked liver dECM bioink in which HepG2 cells were evaluated [321]. However, the use of HepG2 cells in liver tissue engineering is limited by the fact that they do not fully reflect the normal function of hepatocytes. Researchers have also developed a dECM bioink as a platform for hepatocellular carcinoma progression studies using digital light processing methods [323]. Mao et al. prepared a liver-specific bioink suitable for digital light processing by combining GelMA with liver dECM, which was wrapped in hiHep cells to form a cell-loaded bioink (Fig. 9B) [324]. Research on dECM-based liver tissue engineering is rapidly developing, and a number of milestones have been achieved. Therefore, new therapies for the treatment of liver diseases in clinical settings are expected in the future.

### 4.7 Urinary system

Kidneys, which are responsible for maintaining the water balance of the body and excreting waste products, contain glomeruli for tubular devices and ultrafiltration for reabsorption [325,326]. Kidney transplantation is regarded as the most effective treatment for patients with end-stage renal disease [327]. Currently, dECM stents provide a treatment for patients with kidney disease, retaining both the structure and composition of the kidney ECM and its unique functions such as reabsorption, secretion, and filtration [[328], [329], [330]].

**Fig. 10:**
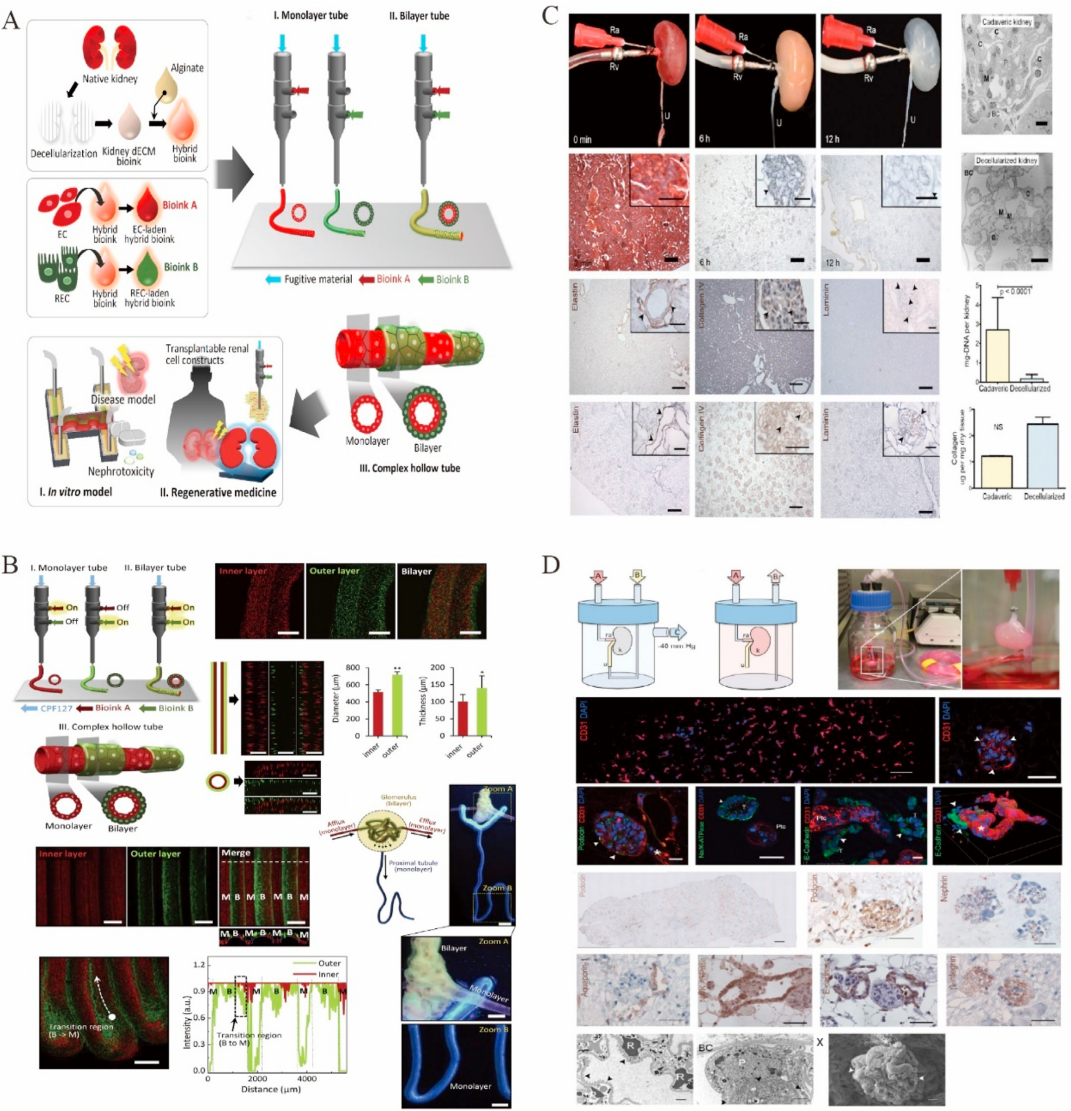
Functional decellularized extracellular matrix in the urinary system repair. (A) Diagram of kidney dECM bioink research and functional validation [337]. (B) Monolayer and bilayer structures are printed coaxially in intricate hollow tubes. Adapted reprinted with permission from Ref. [337] (License number:5,450,780,535,429). (C) Gross views and immunohistochemical staining images of rats before and after whole kidney perfusion decellularization. Adapted reprinted with permission from Ref. [332] (License number:5,450,781,044,751). (D) Apparatus and histological images of recellularization and culture of decellularized rat kidney Adapted reprinted with permission from Ref. [332] (License number:5,450,781,044,751).

**Fig. 11:**
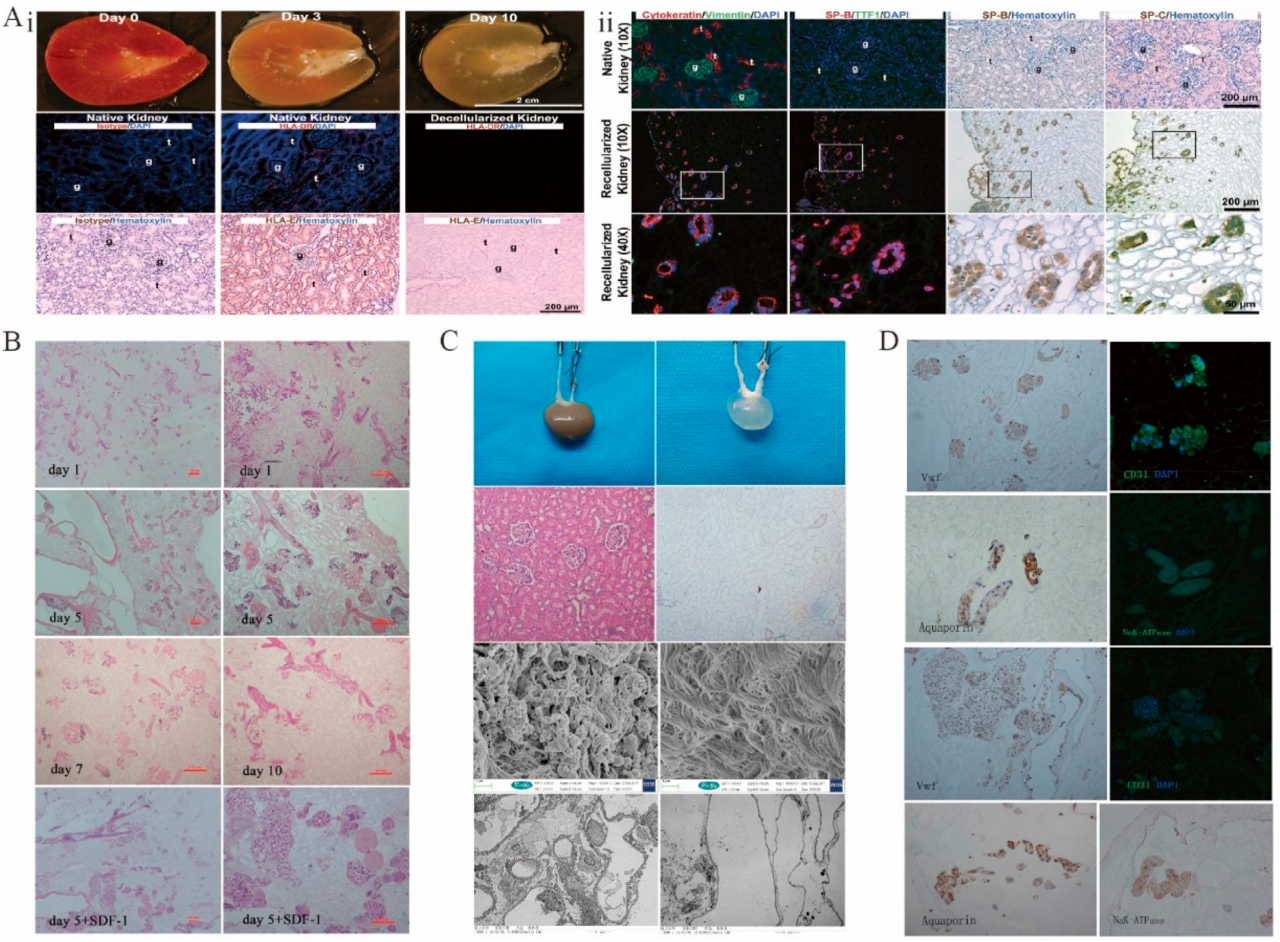
Functional decellularized extracellular matrix in the urinary system repair. (A) Decellularization and in-cellularitytion of the rhesus monkey kidney. (i) Decellularization of rhesus monkey kidney sections. (ii) Immunohistochemistry of decellularized kidney scaffolds recellularized with undifferentiated human embryonic stem cells. Adapted reprinted with permission from Ref. [334], based on CC BY License. (B) H&E staining after kidney recellularization [335]. (C) Perfusion decellularization and histology of whole rat kidneys [335]. (D) Day 5: recellularized kidney immunohistochemistry and immunofluorescence stains following SDS decellularization and whole organ culturing. Adapted reprinted with permission from Ref. [335] (License number:5,450,790,149,095).

Kidneys can be decellularized to <10% residual DNA content using enzymatic digestion [331] or 1% SDS [332]. Since the first *in situ* bioengineered kidney transplant in rodents was reported in 2013 (Fig. 10C and D) [332], a growing number of studies have focused on maintaining kidney-specific growth factors, increasing vascular integrity, and reducing decellularization time [110,327,333]. Renal dECM with preserved glomerular, tubular, and vascular structures has been used as a platform for renal regenerative therapies, as exemplified by embryonic stem cells grown on renal dECM (Fig. 11A) [334] and generated from adipose tissue stem cells (Fig. 11B–D) [335]. Abhigyan et al. developed a new platform by decellularizing fibroblasts grown on surfaces with macromolecular crowding to mimic the natural kidney microenvironment, and human immortalized foot cells cultured on this platform showed superior viability and metabolic activity for up to 28 days [336]. To address complex renal tubular structures such as bilayer glomeruli or proximal renal tubules positioned side-by-side with blood vessels, a 3D microfluidic renal tubular tissue chip was designed for drug screening and regenerative medicine applications (Fig. 10A and B) [337]. Although a kidney-resembling structure has been reconstructed *in vitro*, its function has not been demonstrated *in vivo* [15]. Further exploration is needed before functionalized decellularized matrices can be used for clinical kidney repair.

### 4.8 Reproductive system

**Fig. 12:**
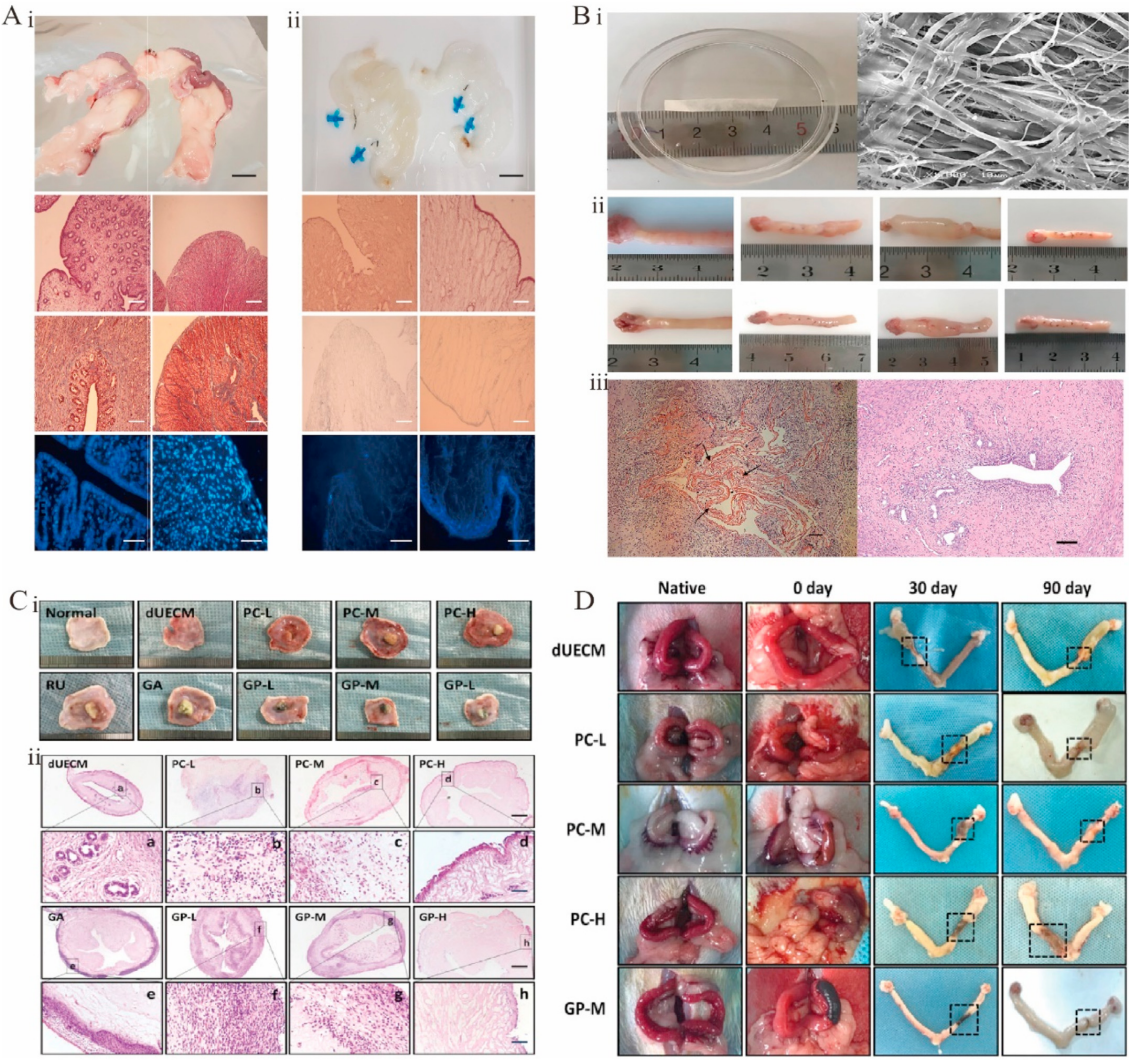
Functional decellularized extracellular matrix in reproductive system repair. (A) Rabbit uterine decellularization. (i) Rabbit uterus before decellularization, and its tissue H&E, Masson's trichrome, and DAPI stain. (ii) After decellularization of the rabbit uterus and its tissue H&E, Masson's trichrome, and DAPI stain, it can be seen that the cells have been largely removed. Adapted reprinted with permission from Ref. [347], based on CC BY NC ND License. (B) UBM repairs the uterine horn in rats. (i) Appearance and structure of UBM. (ii) Appearance of the uterine horns in the experimental groups; (iii) Complete degradation of UBM material in the experimental groups after 4 weeks. Adapted reprinted with permission from Ref. [349], based on CC BY License. (C) Non-cross-linked and cross-linked dUECM subcutaneous embedding experiments were performed 14 days later for (i) histological observation (ii) and HE staining. Adapted reprinted with permission from Ref. [350], based on CC BY License.(D) Morphological observation of cross-linked dECM in the reconstructed uterus of rats *in vivo*. Adapted reprinted with permission from Ref. [350], based on CC BY License. Abbreviations: UBM: Urinary bladder matrix. dUECM:decellularized rabbit uterus matrix.

Despite the numerous advances in the treatment of female infertility, endometriosis, endometrial cancer, and serious uterine adhesions still require hysterectomy, which may lead to uterine dysfunction or infertility [338]. Infertility is still a widespread problem [339]. Uterus transplantation, which has shown great potential in infertility treatment, has become an effective option for these patients [340,341]. Induction of angiogenesis is a clinically effective strategy for the treatment of thin endometrium [342]. As a tissue scaffold, functionalized decellularized tissue may provide follicular growth support for the development of new infertility treatments [343,344], including the treatment of uterine disease–caused infertility [345]. Recent studies have confirmed that biomaterials with tissue-specific ECM characteristics can promote uterine regeneration and support pregnancy in severely damaged uterine models [346]. Functionalized decellularized tissue has also been used to repair endometrial loss, and the current dECM selection for repairing endometrial damage is focused on three tissue sources, namely endometrial tissue–derived dECM (most specific) (Fig. 12A) [347], amniotic membrane–derived dECM from fetal placenta (most widely used) [348], and urinary tissue–derived dECM (structurally similar to uterine endometrial tissue–derived dECM) (Fig. 12B) [349]. Although urinary tissue–derived dECM has the lowest DNA content and maintains the bioactive components to a considerable extent, the tissue obtained after decellularization does not maintain the tubular structure and is less compact. Both *in vitro* and *in vivo*, urinary tissue-derived dECM showed a significantly low tensile strength and modulus of elasticity while featuring a rapid degradation rate, which led to the failure of uterine regeneration due to insufficient support strength (Fig. 12C and D) [350]. After a month, the dECM uterus had completely broken down, but the place where the uterus had been cut had not healed. Poor mechanical properties result in the deformation of the transplanted uterus and prevent the maintenance of macroscopic (especially fine tissue) structures, while rapid stent degradation prevents tissue regeneration and reconstruction as well as endometrial cell growth and extension. The mechanical properties of urinary tissue–derived dECM can be significantly enhanced by crosslinking natural genistein and proanthocyanidins, which also allows one to decrease the rate of enzymatic degradation and achieve significant cell permeability, low immunoreactivity, and excellent biocompatibility [350]. In rats with an annulated uterus, the uterus was completely regenerated after 90 days, whereas a decellularized rabbit uterine matrix was mostly degraded. However, the most suitable concentration for crosslinking has further drawbacks.

### 4.9 Functional dECM in other systems

#### 4.9.1 Muscle

**Fig. 13:**
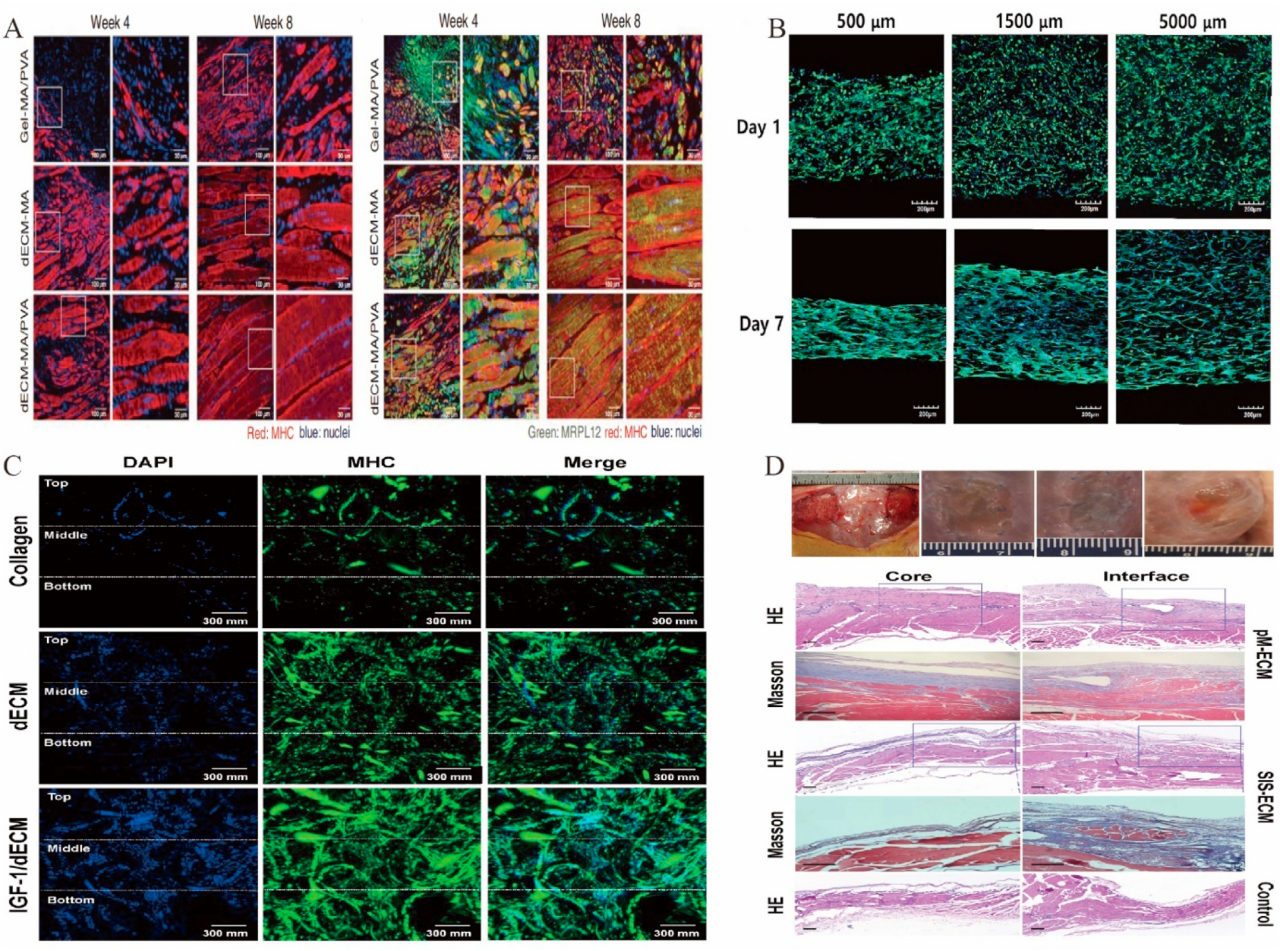
Functional decellularized extracellular matrix in Muscle repair. (A) MHC-stained images of TA muscle taken at 4- and 8-weeks post-implantation (red), demonstrating new muscle fiber formation. Dual immunofluorescence imaging of MRPL12 (green) and MHC (red), indicating that hMPC is differentiated. Adapted reprinted with permission from Ref. [354] (License number: 5,450,791,471,088). (B) Confocal fluorescence images of muscle structures at 500, 1500, and 5000 ​m line width on days 1 and 7; green indicates F-actin (oncoprotein) and blue indicates the nucleus (Dpi). Adapted reprinted with permission from Ref. [357](License number: 5,450,800,377,648). (C) After seven days, collagen, dECM, and IGF-1/dECM scaffolds were stained for MHC (blue ​= ​nuclei; green ​= ​MHC). IGF-1/dECM had higher MHC expression than other groups, indicating more myotubes. Adapted reprinted with permission from Ref. [352] (License number: 5,450,800,774,816). (D) Rat abdominal wall repair: gross and morphological observations Left: surgical repair using perfusion skeletal muscle ECM (pM-ECM); right: control. In pM-ECM-healed defects, samples without seroma had neo-muscle fibres along the margin and in the middle. Adapted reprinted with permission from Ref. [351] (License number: 5,450,801,213,501)..

According to the World Health Organization, 1.71 billion people worldwide were affected by skeletal muscle disease in 2021. Regenerative medicine offers a new way of thinking about this class of diseases. The culturing and differentiation of C2C12 myogenic cells on dECM scaffolds resulted in the formation of myofibers with large diameters, high fusion indices, and great metabolic capacity (Fig. 13D) [351]. Compared to native skeletal muscle, decellularized rabbit skeletal muscle retained approximately 50% of collagen, 74% of elastin, and 83% of GAG (Fig. 13C) [352]. The morphology of the dECM scaffold usually affects the alignment of skeletal muscle cells. Myogenic cells from C2C12 mice were inoculated on directed and nondirected dECM scaffolds, and myogenic cell arrangement, myotube formation, and myogenic gene expression were found to be increased in the former case [353]. A polyvinyl alcohol–dECM bioink produced by photocrosslinking was used to 3D print skeletal muscle structures. Well-aligned printed skeletal muscle structures significantly expressed MHC+, reduced muscle fibers, and increased myofiber formation, producing greater tonicity (Fig. 13A) [354]. These studies demonstrate the regulatory role of the topographical arrangement of the dECM in directing myogenesis, which allows for targeted cell growth and enhanced muscle production *in vitro* and muscle regeneration *in vivo*.

The morphology of skeletal muscle–derived dECM can be regulated at the micron or nanometer level to guide cell behavior and muscle differentiation. However, the morphology and scale that are most effective in achieving optimal cellular responses remain unclear [355]. Microscale cell-loaded dECM muscle structures were 3D printed with linewidths of 300–1000 ​μm [356] to afford a structure supporting the formation of densely arranged myotubes and featuring a high cell survival rate. Such muscle structures were implanted into defects in rat tibial anterior volumetric muscle loss injuries and achieved the regeneration of new muscle and minimal fibrosis compared with unpatterned controls. C2C12-loaded scaffolds with 500–5000-μm fibers were fabricated by 3D bioprinting, with cells cultured in the 500-μm patterned scaffolds showing the greatest orientation extent, elongation, and mechanical strength (Fig. 13B) [357].. In summary, the abovementioned studies highlight the importance of porosity in the fabrication of skeletal muscle–derived dECM. Excessive porosity leads to reduced structural integrity, while inadequate porosity increases the risks of hypoxia and cell death [358]. In addition, mechanical properties are a key factor to consider in skeletal muscle tissue engineering [359]. To mimic muscle structure, researchers used a dECM bioink to 3D print skeletal muscle scaffolds that exhibited a stiffness of 12 ​± ​3 ​kPa *in vitro* and supported myogenic differentiation, myogenic cell proliferation, and myotube formation [357]. Porous/fibrous scaffolds were fabricated by hot stretching and had a stiffness (12.4 ​± ​3.5 ​kPa) close to that of natural muscle (11.5 ​± ​1.3 ​kPa) [360]. Despite the numerous advances in the 3D printing of skeletal muscle–derived dECM, the commercialization of dECM using rigorous bioprinting techniques has not yet been achieved [360]. Currently, substantial research and development are required before such methods become standard clinical practice.

#### 4.9.2 Stroma in cornea

**Fig. 14:**
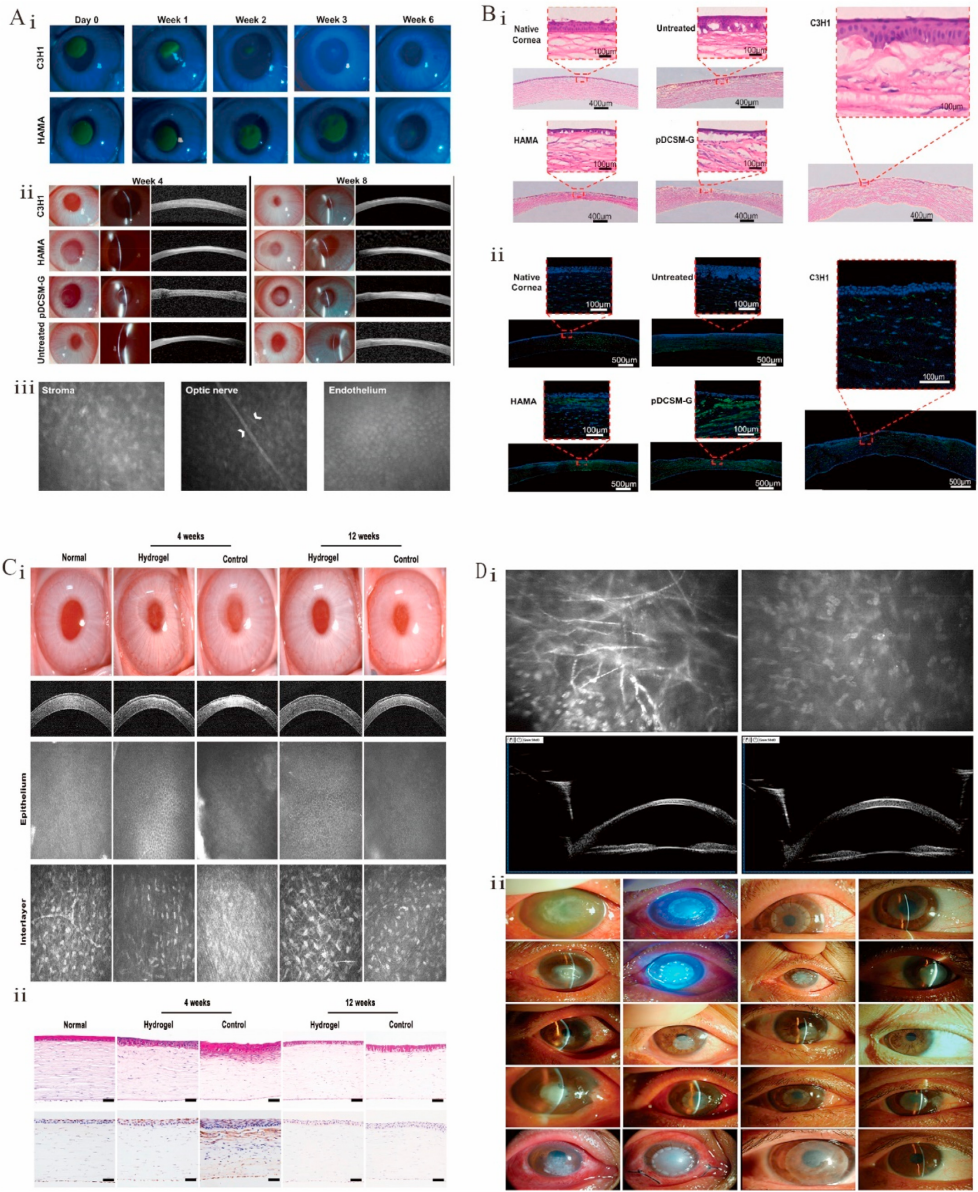
Functional decellularized extracellular matrix in the cornea. (A) Postoperative observation of the hydrogel treated corneas (i) Typical images of fluorescein and cobalt blue stains in rabbit experiment eyes. (ii) Slit lamp and anterior segment optical coherence to-mography images. (iii) Confocal micrographs [377]. (B) Eight weeks after surgery, histological examination of the hydrogel-treated rabbit corneas. (i) H&E staining results. (ii) Images of immunofluorescence stained using biomarkers α-SMA (green) and DAPI (blue). Adapted reprinted with permission from Ref. [377], based on CC BY License. (C) Application of CMC/NHS cross-linked decellularized porcine corneal hydrogels. (i) Postoperative observation and (ii) histological analysis of CMC/NHS cross-linked decellularized porcine cornea hydrogels. Adapted reprinted with permission from Ref. [378](License number: 5,450,810,577,129). (D) Comparison of corneas before and after surgery. (i) In vivo confocal microscopic and ultrasound biomicroscopic images of patients. (ii) Corneal slit lamp micrograph before and after surgery. Adapted reprinted with permission from Ref. [372](License number:5,450,810,989,732). Abbreviations: CMC/NHS:N-cyclohexyl-N′-(2-morpholinethyl) carbodiimide metho-p-toluene sulfonate/N-hydroxy succinimide. (For interpretation of the references to colour in this figure legend, the reader is referred to the Web version of this article.)

The cornea is the most important refractive element in enabling vision, with cornea damage or infection often resulting in stroma scarring and thinning, which may impair vision and even cause blindness [361]. In such cases, the most common treatment is to partly or fully replace the cornea with human donor tissues [362]. However, as with all transplants, the donor sources are highly restricted, with less than 5% of patients undergoing corneal transplantation [363]. Consequently, the replacement of diseased corneas with synthetic ones has been actively studied as an alternative to conventional corneal tissue transplantation [[364], [365], [366]]. Decellularized cryomilled corneal powder has been used for corneal repair and regeneration [367,368]. Human cornea–derived dECM particles dispersed in a human fibrin sealant were used for anterior corneal stromal reconstruction [369]. The results showed that the incorporation of corneal tissue–derived ECM particles into fibrin hydrogels does not pose toxicity or safety risks and shows great promise as a minimally invasive treatment for superficial corneal epithelial trauma and anterior stromal injury. Decellularized bovine and porcine corneas have been shown to be biocompatible and maintain the optical transparency, mechanical properties, and structural integrity of the original cornea [370,371]. Decellularized porcine corneas have been clinically used as tissue-engineered scaffolds for allogeneic corneal transplants (Fig. 14D) [372]. The derived hydrogels allowed rapid corneal epithelialization [373], although the achieved transparency and mechanical strength were low. Crosslinking is an effective way to increase mechanical strength, e.g., the *N*-cyclohexyl-*N*’-(2-morpholinoethyl) carbodiimide metho-*p*-toluene sulfonate/*N*-hydroxysuccinimide (CMC/NHS) crosslinker increases the toughness of decellularized porcine cornea–derived hydrogels without affecting their biological activity [374,375]. In addition, a high secretion of collagenase and metalloproteinases was observed during stromal repair [376], which may prevent the long-term retention of decellularized corneal hydrogels on corneal defects. A hybrid hydrogel composed of hyaluronic acid methacrylate (HAMA) and porcine decellularized corneal stromal matrix (pDCSM) was produced by crosslinking and used to directly fill corneal defects of various shapes, showing excellent adhesion properties and cell penetration performance owing to its microporous 3D network structure [377]. This system also exhibited low swelling, slow degradation, enhanced physical characteristics, and cornea-matched transparency, and was capable of withstanding ultrahigh intraocular pressure. Histological analysis showed that the corneal stroma filled with the pDSCM pregel solution and the 2% (w/v) HAMA pregel solution mixed in a 3:1 ​vol ratio underwent ordered alignment (Fig. 14A and B) [377]. The corneal model was used with CMC/NHS crosslinked with pDCSM-G, which promoted epithelial recovery and stromal regeneration (Fig. 14C) [378]. Although cornea-derived dECM hydrogels have been used for corneal repair, the design of cells containing functional corneal structures and maintaining transparency remains difficult [21].

## 5 Conclusion and outlook

With the advent of numerous decellularization techniques, dECM has evolved from simple tissue scaffolds to whole-organ scaffolds, while the recent emergence of 3D printing technologies exploiting dECM-based bioinks enables the fabrication of more complex whole-organ scaffolds. In this review, we described the composition and role of the ECM in stem-cell differentiation and summarized the advantages and disadvantages of existing decellularization techniques and methods for the refunctionalization of decellularized scaffolds. The applications, development progress, and challenges faced by functionalized dECM scaffolds in different tissues and organs were discussed, as exemplified by the repair of cartilage, skin, nerve, and muscle injuries and the transplantation or regeneration of whole organs such as liver, heart, uterus, kidneys, and lungs. Some of these materials have been successfully used in both animal models and clinical applications, as exemplified by human dermal dECM–based products for treating ligament and tendon injuries, namely Allopatch HD™ (MTF sports drug) and GraftJacket® (Wright Medical), and the cell-free pericardium-based heart valve product CardioCel® (Admetus IHS Inc.) (Table 3).

**Table 3:** Commercialized products of decellularized extracellular matrix.

Commercial options	Materials	Applications
Allderm®	Human allogeneic dermal decellularized matrix	Repair of skin wounds
OASIS®	Decellularized stroma of the porcine small intestine submucosa (SIS)	Repair of skin wounds
Prima™ Plus	Decellularized heart valves of the porcine	Repair of heart valves
CardioCel®	Decellularized heart valves of cattle	Repair of heart valves
GraftJacket®	Human allogeneic dermal decellularized matrix	Repair of tendon and ligament injuries
Allopatch HD™	Human allogeneic dermal decellularized matrix	Repair of tendon and ligament injuries
AxoGuard®	Decellularized stroma of the porcine small intestine submucosa	Used as a nerve connector
Human Acellular Vessel	Decellularized matrix of human allogeneic blood vessels	Repair of blood vessels（Phase 3 clinical trial）

Although the dECM technology has achieved long-term advances in the field of tissue engineering, several persistent challenges remain, e.g., (1) the need for (i) standardized decellularization protocols and characterization methods to better preserve the biological and mechanical properties of the ECM and (ii) more sensitive testing of residual cellular components and toxic products. (2) Moreover, better sterilization methods, especially those for virus eradication, should be developed while minimizing their impact on the mechanical and biological characteristics of tissue materials. (3) The optimization of the dECM functionalization protocol should be continued. The crosslinking of loading factors, bioactive molecules, and/or mechanical modifications contribute to tissue repair and regeneration. Recellularization processes must consider clinically relevant sources of regenerable cells, inoculation methods (at this stage, the most common delivery techniques are cell infusion, cell injection, or simple surface application [5]), and bioreactors with physiologically relevant organ culture conditions. (4) The diversity and complexity of tissues and organs need to be considered, as different tissues and organs have different structures, which complicates the production of optimal bionic materials derived from the dECM. dECM-derived bioinks offer a viable approach to 3D printing, although further in-depth studies are required. (5) The optimization of hematologic reconstitution and thrombosis and inflammatory response reduction are essential for successful tissue and organ regeneration. Currently, thrombosis remains a challenge when whole-heart dECMs are used *in vivo* [5]. Moreover, DNA with length of <200 bp does not cause significant inflammatory responses [379], which should be taken into account in future studies. (6) Although the ideal decellularization protocol removes all immunogenicity from porcine tissues, the risk of using animal sources in humans still exists [21]. The assessment of immunogenicity and biocompatibility is critical for final health-authority approval, commercial viability, and clinical application.

Despite the numerous challenges associated with the clinical use of refunctionalized dECMs for the repair of various organs and tissues in the human body, these materials are considered to be low immunogenic scaffolds for tissue/organ repair providing an environment for stem-cell differentiation and growth and featuring properties that can be optimized by crosslinking various natural tissue-engineering materials. However, these materials should be further evaluated in *vivo* animal experiments while considering the differences between animal models and humans. In conclusion, dECM refunctionalization is a promising solution for regenerative medicine and tissue engineering.

## Declaration of competing interest

The authors declare that they have no known competing financial interests or personal relationships that could have appeared to influence the work reported in this paper.

## Data Availability

No data was used for the research described in the article.
